# PPM1A Regulates Antiviral Signaling by Antagonizing TBK1-Mediated STING Phosphorylation and Aggregation

**DOI:** 10.1371/journal.ppat.1004783

**Published:** 2015-03-27

**Authors:** Zexing Li, Ge Liu, Liwei Sun, Yan Teng, Xuejiang Guo, Jianhang Jia, Jiahao Sha, Xiao Yang, Dahua Chen, Qinmiao Sun

**Affiliations:** 1 State Key Laboratory of Biomembrane and Membrane Biotechnology, Chinese Academy of Sciences, Chaoyang District, Beijing, China; 2 State Key Laboratory of Reproductive Biology, Institute of Zoology, Chinese Academy of Sciences, Chaoyang District, Beijing, China; 3 State Key Laboratory of Proteomics, Genetic Laboratory of Development and Diseases, Institute of Biotechnology, Beijing, China; 4 State Key Laboratory of Reproductive Medicine, Department of Histology and Embryology, Nanjing Medical University, Nanjing, China; 5 Markey Cancer Center, Department of Molecular and Cellular Biochemistry, The University of Kentucky College of Medicine, Lexington, Kentucky, United States of America; University of Southern California, UNITED STATES

## Abstract

Stimulator of interferon genes (STING, also known as MITA and ERIS) is critical in protecting the host against DNA pathogen invasion. However, the molecular mechanism underlying the regulation of STING remains unclear. Here, we show that PPM1A negatively regulates antiviral signaling by targeting STING in its phosphatase activity-dependent manner, and in a line with this, PPM1A catalytically dephosphorylates STING and TBK1 *in vitro*. Importantly, we provide evidence that whereas TBK1 promotes STING aggregation in a phosphorylation-dependent manner, PPM1A antagonizes STING aggregation by dephosphorylating both STING and TBK1, emphasizing that phosphorylation is crucial for the efficient activation of STING. Our findings demonstrate a novel regulatory circuit in which STING and TBK1 reciprocally regulate each other to enable efficient antiviral signaling activation, and PPM1A dephosphorylates STING and TBK1, thereby balancing this antiviral signal transduction.

## Author Summary

Innate antiviral immunity is essential for the host defense system that rapidly detects and eliminates invading viruses. STING, an endoplasmic reticulum (ER)-associated protein, plays important roles in the activation of type I IFN in response to DNA virus infection. Whereas excessive activation of STING can potentially cause lethal inflammatory diseases, STING activity must thus be precisely controlled to ensure the proper antiviral signaling transduction. However, the mechanisms of how STING activation is regulated are not fully understood. In this study, we find that PPM1A physically interacts with STING and negatively regulates STING-mediated antiviral signaling. Mechanistically, we find that PPM1A functions as a phosphatase that targets both STING and TBK1 for their dephosphorylation. Moreover, our study demonstrates that while TBK1 enhances STING aggregation in a kinase activity-dependent manner, PPM1A suppresses STING aggregation by dephosphorylating both STING and TBK1. Collectively, our study not only reveals that STING and TBK1 reciprocally regulate each other’s activity to elicit antiviral signaling, but also shows that PPM1A antagonizes STING aggregation by targeting both STING and TBK1, thereby maintaining proper antiviral responses.

## Introduction

Innate immunity is the first line of the host defense system against invading microorganisms, including viruses. The detection of conserved microbial molecules, known as pathogen-associated molecular patterns (PAMPs), by the host cells involves multiple pattern recognition receptors (PRRs). Nucleic acids derived from viruses act as PAMPs to trigger the innate immune response when they are recognized by PRRs, leading to the production of a variety of cytokines, including type I interferons (IFNs).

Host cells have evolved multiple sensing systems to recognize PAMPs during viral infection, in a manner that depends on the cellular location and ligand specificity. For example, Toll-like receptors (TLRs) such as TLR3, TLR7, and TLR8 can detect different species of RNA viruses in the endosomal compartment [1], whereas the RIG-I-like receptors, RIG-I and MDA5 function as cytoplasmic sensors of viral RNA [2,3]. In response to RNA viral infection, RIG-I and MDA5 form a complex in the cytosol with the adaptor protein MAVS [4] (also known as IPS-1 [5], VISA [6], and CARDIF [7]), efficiently inducing a conformational switch of MAVS that causes MAVS aggregation and activation [8]. Activated MAVS then recruits TANK-binding kinase 1 (TBK1) and the IκB kinase (IKK) complex to activate transcription factors interferon regulatory factor 3/7 (IRF3/7) and NF-κB respectively, which coordinately induce the production of type I IFNs and elicit the innate immune response.

Nucleic acids from DNA viruses also induce potent innate immune responses. A number of cytoplasmic DNA sensors, including AIM2 [9–12], DAI [13], IFI16 [14], DDX41 [15], and cGAS [16], have recently been identified. STING (also called MITA [17], and ERIS [18]) is an endoplasmic reticulum (ER)-associated molecule that acts as an adaptor for the activation of type I IFN in response to cytosolic nucleic acid ligands. Upon activation, STING interacts with TBK1 and activates transcription factor IRF3 to induce the production of type I interferon genes. Although STING is critical for the protection of the host against the invasion of DNA pathogens, its excessive activation can potentially cause lethal inflammatory diseases [19,20]. Therefore, STING activity must be precisely controlled to ensure a proper innate immune homeostasis in infected host cells. Several factors, such as RNF5 [21], Atg9a [22], ULK1 [23], and NLRC3 [24], have recently been shown to suppress the activation of STING. A previous study [25] suggested that, whereas STING induces TBK1 activation, STING itself can be phosphorylated and polymerized that further contributes to IRF3 activation, and the phosphorylated and polymerized STING thus probably forms a scaffold platform to recruit TBK1 and IRF3. These findings indicate an intriguing feed-forward mechanism, *via* the regulation between STING and TBK1, which efficiently triggers innate immune antiviral signaling.

Previous studies have suggested two distinct models regarding phosphorylation of STING for regulating its function. Konno et al. found that ULK1 phosphorylated STING at S366 for its degradation, and thereby suppressing the downstream IRF3 signaling activity [23]. However, a most recent study reported that STING phosphorylation at S366 by TBK1 is required for direct IRF3 recruitment and activation, rather than for STING degradation [26]. Thus, the issue of how STING phosphorylation contributes to STING signaling activity has remained largely unclear.

In an effort to understand the molecular mechanism underlying the regulation of STING in detail, we performed yeast two-hybrid screening and identified protein phosphatase, Mg^2+^/Mn^2+^-dependent, 1A (PPM1A), a member of the PP2C family of serine/threonine (Ser/Thr) protein phosphatases, as a STING-interacting protein. We demonstrated that PPM1A negatively regulates antiviral signaling by targeting STING for dephosphorylation in a phosphatase activity-dependent manner. We also found that PPM1A directly dephosphorylates STING and TBK1 in *in vitro* assays. Importantly, we provide evidence that whereas TBK1 promotes STING aggregation in a phosphorylation-dependent manner, PPM1A antagonizes STING aggregation by dephosphorylating both STING and TBK1, emphasizing that phosphorylation is a crucial step for efficient STING activation. Together, our findings identify a novel regulatory circuit in which STING and TBK1 reciprocally regulate one another to elicit antiviral signaling, whereas PPM1A dephosphorylates STING and TBK1, thereby balancing antiviral signal transduction.

## Results

### Identification of PPM1A as a STING-interacting protein

**Fig 1:**
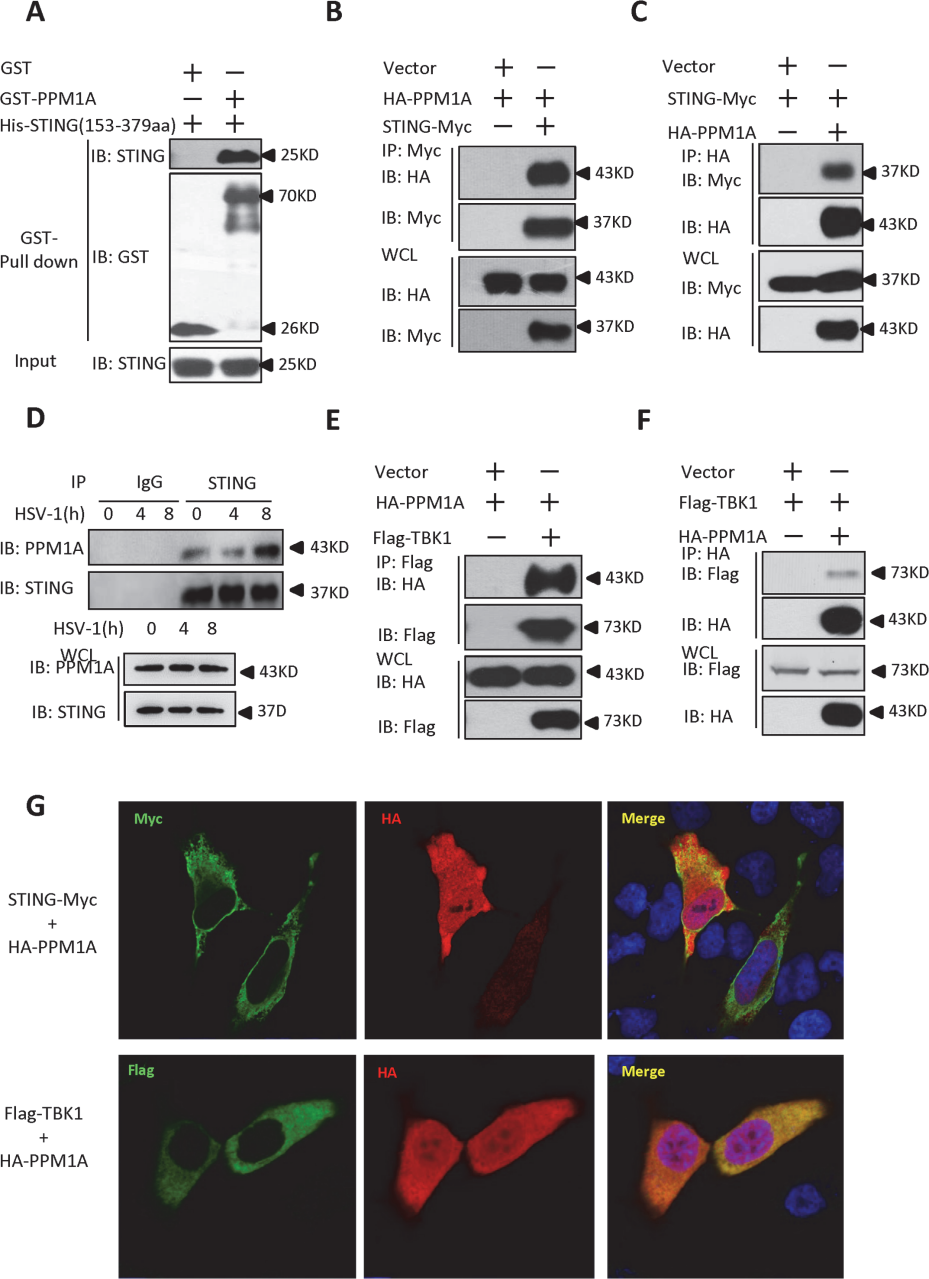
Identification of PPM1A as a protein that interacts with STING. (A) Purified GST–PPM1A interacted with His–STING (amino acids 153–379) in an *in vitro* protein-binding assay. Purified His–STING (amino acids 153–379; 1 μg) was incubated with purified GST–PPM1A (1 μg) or GST (1 μg), and then pulled down with glutathione–Sepharose beads. Western blots were performed to analyze the presence of His-tagged STING and GST-tagged PPM1A proteins. (B, C) Epitope-tagged PPM1A and STING interacted with each other in HEK293 cells. HEK293 cells were transfected with the indicated DNA plasmids. At 24 h post-transfection, the lysates were immunoprecipitated with anti-Myc beads (B) or anti-HA beads (C), followed by immunoblotting analysis with the indicated antibodies. WCL (bottom), expression of exogenous proteins in whole-cell lysates. (D) More PPM1A proteins were found to be present in the STING immunoprecipitates after HSV-1 virus infection. THP-1 cells were infected with or without HSV-1 at 5 MOI for the indicated times. The cells were lysed and immunoprecipitated with rabbit anti-STING antibody or an IgG control antibody. Immunoprecipitates were analyzed by immunoblotting with the anti-PPM1A and anti-STING antibodies. Expression levels of endogenous PPM1A and STING were detected with an immunoblotting analysis. (E, F) Epitope-tagged PPM1A and TBK1 interacted in HEK293 cells. Transfection and co-immunoprecipitation experiments were performed as in B and C, except that Flag-TBK1 expression vector was used instead of STING-Myc. (G) PPM1A is co-localized with both STING and TBK1 in transfected Hela cells. Hela cells were transfected with the indicated plasmids for 24 h, and then fixed with 4% paraformaldehyde, stained with the indicated antibodies, and observed with confocal microscopy.

To understand the molecular mechanisms underlying the regulation of STING in the antiviral innate immune signaling pathway, we performed a yeast two-hybrid screen to identify STING-interacting factors using the C-terminal fragment of STING (amino acids 153–379) as bait. From this screening, we found that one of the positive clones encoded the full-length PPM1A protein (S1A Fig). PPM1A is a member of the PP2C family of Ser/Thr protein phosphatases. PP2C family members are known as negative regulators of cellular stress-response pathways [27] and are involved in cell-cycle control by dephosphorylating cyclin-dependent kinases [28] and also play roles in NF-κB pathway by dephosphorylating IKKβ and P65 [29,30]. To confirm that PPM1A interacts directly with STING, we performed histidine (His)/glutathione S-transferase (GST) *in vitro* pull-down experiments using recombinant His–STING (amino acids 153–379) and GST–PPM1A purified from bacteria. As shown in Fig. 1A, GST–PPM1A, but not GST control protein, pulled down His–STING (amino acids 153–379). Consistently, His–STING (amino acids 153–379) pulled down GST–PPM1A, but not the GST control protein (S1B Fig). To further determine the interaction under physiological condition, we performed co-immunoprecipitation experiments to examine whether STING physically interacts with PPM1A in cultured mammalian cells. As shown in Fig. 1B and C, epitope-tagged PPM1A and STING reciprocally co-immunoprecipitated with each other in transfected HEK293 cells. Consistent with these results, we found that endogenous PPM1A protein was present in the STING complex in THP-1 cells (Fig. 1D). Of note, we found that the endogenous STING–PPM1A association was even reliably detectable under normal physiological condition. Interestingly, we found that viral infection could increase the association between PPM1A and STING, since an apparent elevation of PPM1A levels were detected in the STING immunoprecipitates at the 8-hour time point post-HSV-1-infection (Fig. 1D). Taken together, our findings suggest that PPM1A interacts directly with STING.

Previous study has shown that PPM1B, another member of the PP2C family of Ser/Thr protein phosphatases, associates with TBK1 and negatively regulates antiviral signaling by antagonizing TBK1 activation through dephosphorylation [31]. Because PPM1A and PPM1B are highly similar at the amino acid level, we tested whether PPM1A also associates with TBK1 and regulates its function in HEK293 cells. To test this possibility, we performed co-immunoprecipitation experiments in HEK293 cells transfected with epitope-tagged PPM1A and TBK1. As shown in Fig. 1E and F, epitope-tagged PPM1A and TBK1 were co-immunoprecipitated in HEK293 cells. Consistent with this observation, our immunostaining assays showed that PPM1A co-localized with both STING and TBK1 in transfected Hela cells (Fig. 1G). On the basis of these observations, we reasoned that PPM1A might have a potential role in regulating STING-mediated antiviral innate immune pathway.

### Overexpression of PPM1A inhibits STING-induced antiviral signaling

**Fig 2:**
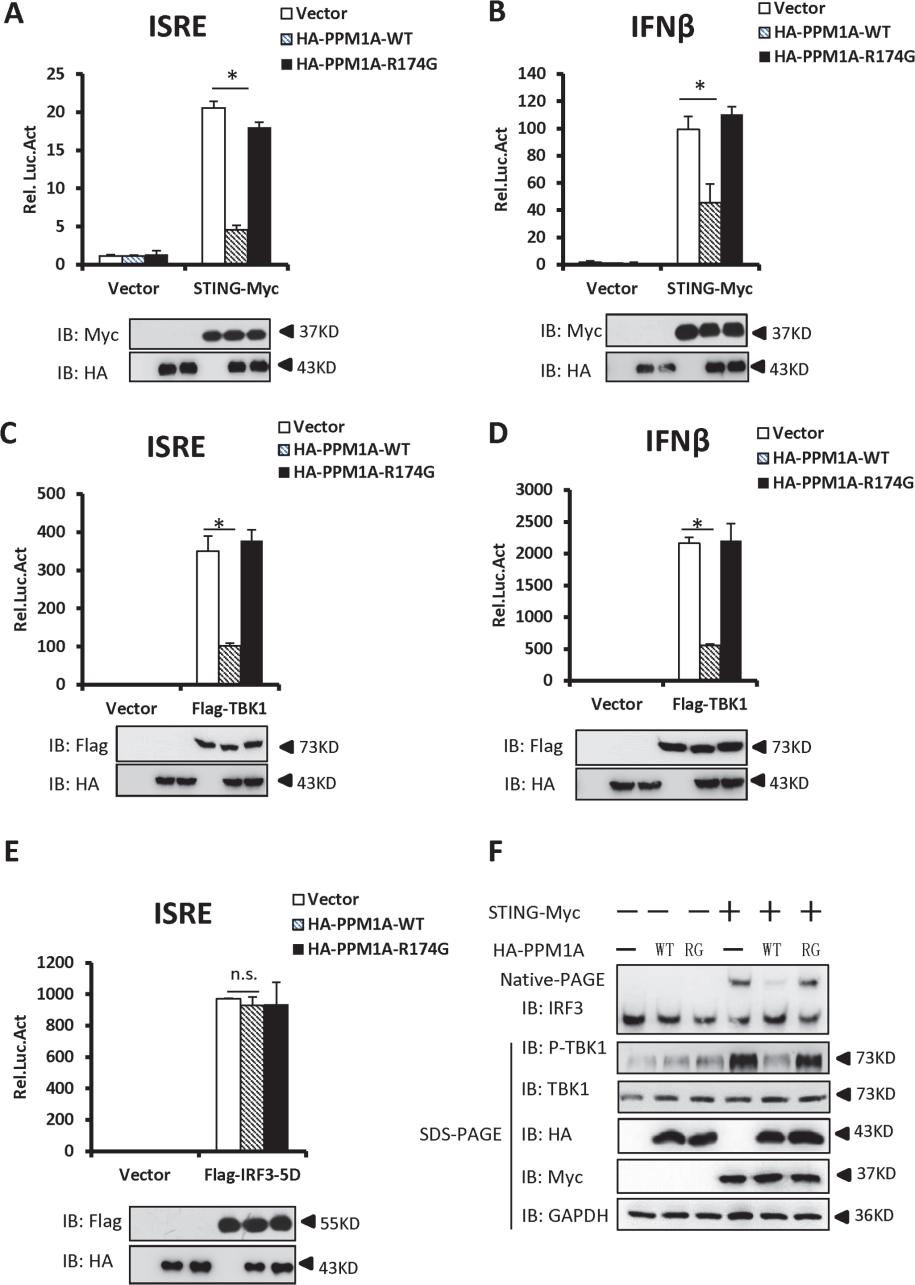
Overexpression of PPM1A inhibits STING-mediated antiviral signaling. (A, B) Overexpression of PPM1A reduced STING-induced activation of the ISRE and IFN-β promoters, which was dependent on the phosphatase activity of PPM1A. HEK293 cells were transfected with the indicated expression plasmids together with luciferase reporter constructs (50 ng) driven by the promoters of genes encoding ISRE (A) or IFN-β (B), and pRSV/LacZ (50 ng) as the internal control. Twenty-four hours after transfection, the cells were lysed for luciferase assays (upper panel) and immunoblotting assays (lower panel). Luciferase activity was measured and normalized to β-galactosidase activity. The results are presented relative to the luciferase activity in the control cells. (C, D) ISRE and IFN-β activation induced by TBK1 was also reduced by PPM1A-WT, but not by PPM1A-R174G. Twenty-four hours after transfection, the cells were lysed for luciferase assays (upper panel) and immunoblotting assays (lower panel). Transfections were performed as in A and B, except that the TBK1 expression vector was used instead of STING-Myc. (E). PPM1A did not affect the IRF3-5D-induced activation of the ISRE promoter. HEK293 cells were transfected with the indicated expression plasmids together with ISRE-luc (50 ng), and pRSV/LacZ (50 ng) as the internal control. Twenty-four hours after transfection, the cells were lysed for luciferase assays (upper panel) and immunoblotting assays (lower panel). (F) PPM1A-WT, but not PPM1A-R174G, significantly reduced STING-induced IRF3 dimerization and TBK1 phosphorylation. HEK293 cells were transfected with expression plasmids as indicated. At 30 h after transfection, the cell lysates were resolved by native gel electrophoresis (upper panel) or SDS-PAGE (lower panels) and analyzed with the indicated antibodies. The data shown in A–E are from one representative experiment of at least three independent experiments (means ± SD of duplicate assays). The two-tailed Student’s *t* test was used to analyze statistical significance. * P < 0.05; n.s., No Significance, versus control groups.

To determine whether PPM1A is involved in the regulation of STING activation, we first tested whether PPM1A contributes to the regulation of STING-induced type I IFN signaling using a cell-based luciferase reporter system. As shown in Fig. 2A and B, the expression of STING significantly activated the promoters of *interferon-stimulated response element* (ISRE) and *IFNβ* in HEK293 cells, whereas the co-expression of PPM1A with STING apparently reduced the STING-induced activation of these promoters, indicating a negative role for PPM1A in regulating STING activity. Because PPM1A acts as a Ser/Thr protein phosphatase, we next tested whether its catalytic activity is required for PPM1A to antagonize STING activation. As shown in luciferase reporter assays, the expression of PPM1A-R174G, a catalytically inactive form of PPM1A [32,33], had apparently lost its capacity to antagonize STING activation (Fig. 2A and B), suggesting that PPM1A suppresses STING function in its phosphatase activity-dependent manner.

We next tested whether PPM1A affects the activation of TBK1 and IRF3. As shown in Fig. 2C and D, the activation of ISRE and IFN-β induced by TBK1, was also suppressed by the expression of wild-type PPM1A, but not by the mutant form, PPM1A-R174G, suggesting that PPM1A phosphatase activity is required for its function in regulating TBK1 activation. In contrast, the expression of PPM1A had no apparent effect on the activation of ISRE induced by IRF3-5D, a constitutively activated form of IRF3 (Fig. 2E), suggesting that PPM1A primarily regulates the signaling upstream of IRF3.

Because the dimerization of IRF3 and TBK1 phosphorylation (at the Ser 172 residue) are important events in the activation of the IFN-β signaling pathway, we tested whether PPM1A modulates STING-mediated IRF3 dimerization and TBK1 phosphorylation. As shown in Fig. 2F, the overexpression of STING led to the dimerization of IRF3 and increase of TBK1 phosphorylation, whereas the co-expression of the wild-type PPM1A, but not mutant PPM1A-R174G, significantly reduced the effects of STING overexpression on IRF3 dimerization and TBK1 phosphorylation. Collectively, our findings suggest that the overexpression of PPM1A antagonizes the STING- and TBK1-induced type I IFN signaling pathway.

### Knockdown of PPM1A potentiates STING signaling

**Fig 3:**
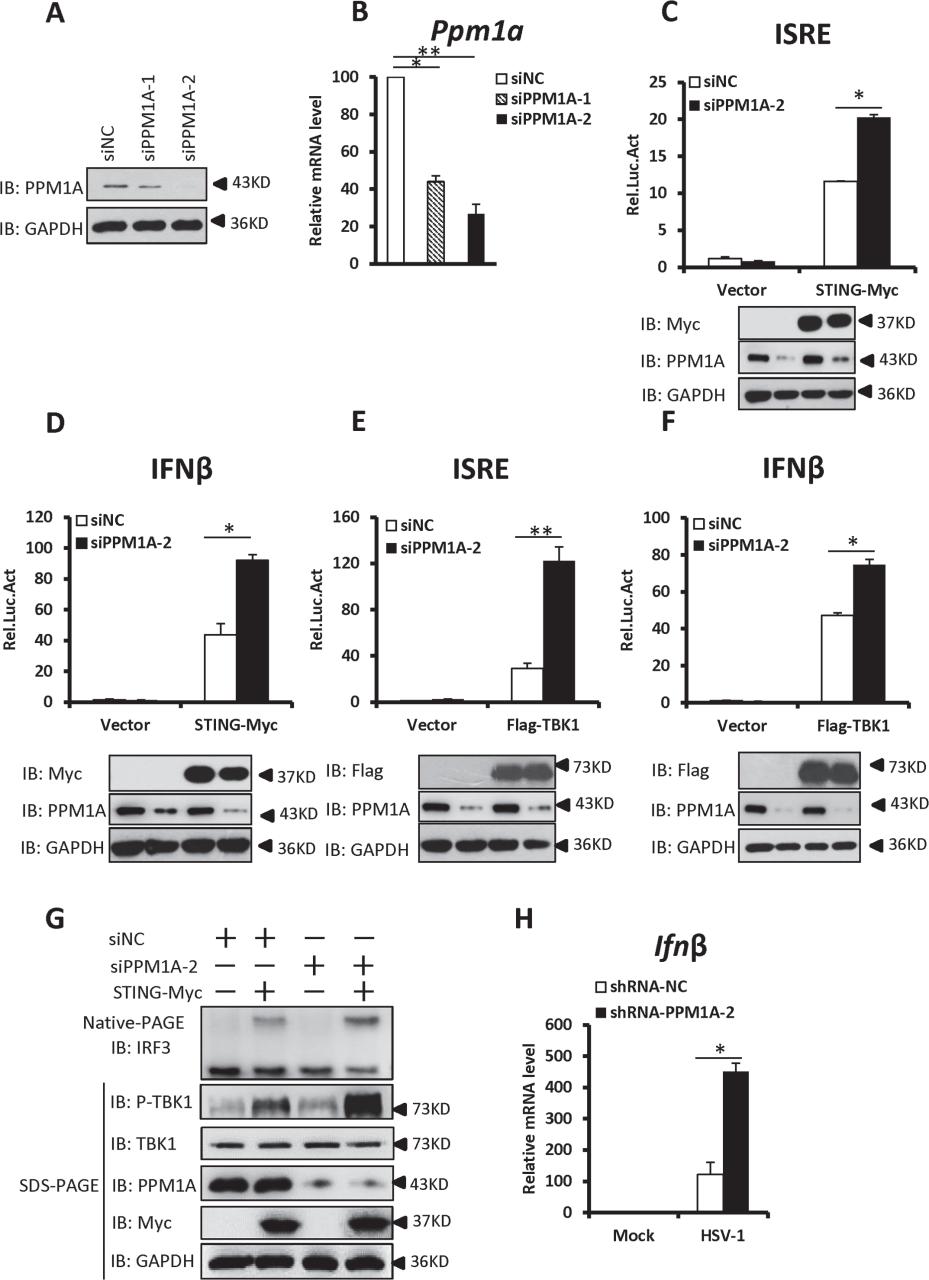
Knockdown of PPM1A potentiates STING signaling. (A, B) Both pairs of PPM1A siRNAs efficiently reduced the levels of endogenous PPM1A at both the protein (A) and mRNA levels (B). HEK293 cells were transfected with siRNA (40 nM) targeting PPM1A or NC (non-targeting control). At 48 h after transfection, the cell lysates were analyzed by immunoblotting (A) or qRT-PCR (B). Data shown in B are the relative levels of *Ppm1a* mRNA in *Ppm1a* knockdown cells to the control cells; the *Ppm1a* mRNA abundance of the control group (siNC) was assigned a value of 100. (C, D) Knockdown of PPM1A potentiated STING-induced activation of the ISRE (C) and IFN-β (D) promoters. HEK293 cells were transfected with siNC (40 nM) or siPPM1A-2 (40 nM). At 48 h after transfection, the cells were transfected with empty vector or STING expression vector together with the reporter plasmids using Lipofectamine 2000 for 20 h. The cells were then lysed for luciferase assays (upper panel) and immunoblotting assays (lower panels). (E, F) Knockdown of PPM1A increased TBK-induced activation of the ISRE (E) and IFN-β (F) promoters. Transfection and luciferase assays were performed as in C and D, except that TBK1 expression vector was used instead of STING expression vector. (G) Knockdown of PPM1A significantly enhanced STING-induced IRF3 dimerization and TBK1 phosphorylation. HEK293 cells were transfected with siNC (40 nM) or siPPM1A-2 (40 nM) for 36 h, and then transfected with empty vector (400 ng) or STING-encoding vector (400 ng) for another 36 h using Lipofectamine 2000. The cell lysates were separated by native gel electrophoresis (upper panel) or SDS-PAGE (lower panel) and analyzed by immunoblotting with the indicated antibodies. (H) Knockdown of PPM1A clearly increased HSV-1-induced transcription of the *Ifnβ* gene. THP-1 cells were infected with a lentivirus targeting PPM1A for knockdown or NC for 72 h and then left uninfected or infected with HSV-1 at a MOI of 5 for 6 h. Cells were lysed and total RNA were isolated for qRT-PCR analysis. Data shown are the relative abundance of *Ifnβ* in the cells treated as indicated, the relative levels of *Ifnβ* in the control cells (siNC, mock) was assigned a value of 1. The data in B–F, and H are from one representative experiment of at least three independent experiments, (mean ± SD of duplicate in C–F or triplicate in B and H). A two-tailed Student’s *t* test was used to analyze statistical significance. * P < 0.05; ** P < 0.01 versus the control groups.

We next investigated whether endogenous PPM1A plays a role in regulating the antiviral response. We first synthesized two different pairs of small interfering RNA (siRNA) oligonucleotides targeting distinct coding regions of the PPM1A mRNA. As shown in Fig. 3A and B, both pairs of siRNA efficiently reduced the expression of endogenous PPM1A at both mRNA and protein levels in HEK293 cells. We selected siPPM1A-2 for the subsequent experiments because it more efficiently down-regulated the expression of PPM1A.

We then determined the effects of PPM1A knockdown on the STING-mediated antiviral signaling. As shown in Fig. 3C–F, knockdown of PPM1A significantly enhanced the STING- or TBK1-mediated activation of the ISRE and IFNβ promoters in HEK293 cells. Consistent with this, the knockdown of PPM1A significantly enhanced STING-induced IRF3 dimerization and TBK1 phosphorylation at S172 (Fig. 3G), but did not apparently influence IRF3-5D-induced ISRE activation (S2A Fig). These data further support the notion that PPM1A negatively regulates STING signaling at the upstream of IRF3.

Given the critical role of STING in protecting the host from DNA pathogens, we next used the THP-1 cell line to test whether PPM1A affects the innate immune signaling induced by DNA virus Herpes simplex virus 1 (HSV-1) by measuring the expression of endogenous *Ifnβ*. As shown in quantitative reverse-transcription polymerase chain reaction (qRT-PCR) assays, short hairpin RNA (shRNA)-mediated PPM1A knockdown clearly increased HSV-1-induced endogenous *Ifnβ* expression (Figs. 3H and S2B), providing further evidence showing the negative role of PPM1A in regulating STING signaling.

### Enhanced STING signaling in *Ppm*1a-deficient cells

**Fig 4:**
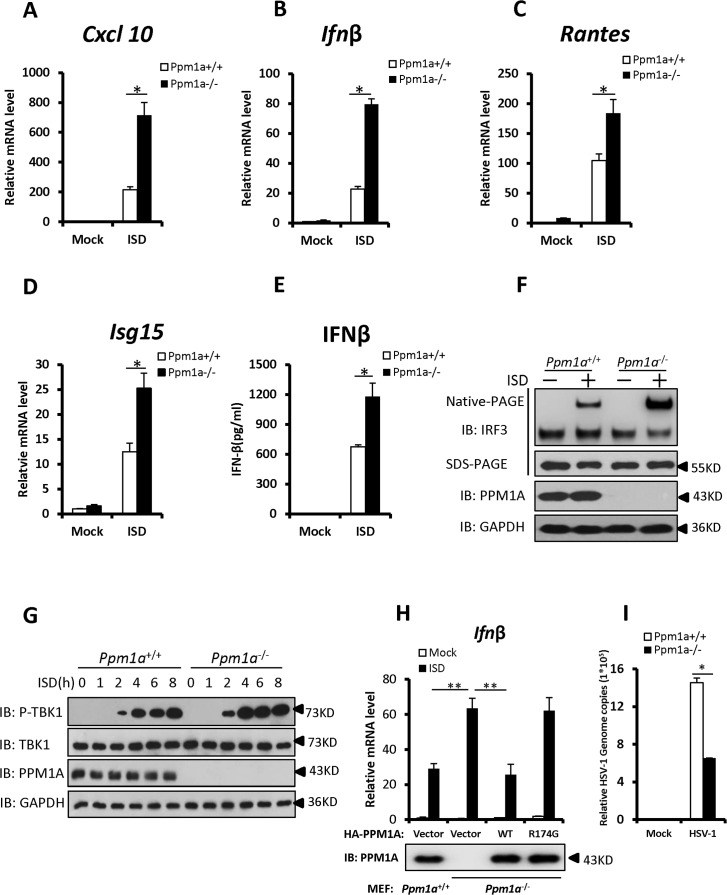
Enhanced STING signaling in *Ppm1a*-deficient cells. (A–D) PPM1A deficiency enhanced the transcriptional levels of antiviral genes. Primary *Ppm1a* ^*+/+*^ and *Ppm1a* ^–/—^MEFs were transfected with ISD (2 μg/ml) for 6 h, followed by qRT-PCR analysis. The transcriptional levels of *Cxcl10* (A), *Ifnβ* (B) *Rantes* (C), and *Isg15* (D) were examined, the relative levels of the indicated mRNA in the control cells (*Ppm1a* ^*+/+*^, mock) was assigned a value of 1. (E) The production of IFN-β protein induced by ISD was increased in *Ppm1a* ^-/-^ MEFs. Primary *Ppm1a* ^+/+^ and *Ppm1a* ^-/-^ MEFs were left untreated or transfected with ISD (2 μg/ml) for 24 h, and then the supernatants were collected for measurement of IFN-β protein by ELISA.(F) ISD-induced IRF3 activation was increased in *Ppm1a* ^*–/—*^MEFs. Primary *Ppm1a* ^+/+^ and *Ppm1a* ^*–/—*^MEFs were left untreated or transfected with ISD (2 μg/ml) for 4 h. The cell lysates were separated by native gel electrophoresis (upper panel) or SDS-PAGE (lower panels) and analyzed by immunoblotting with the indicated antibodies. (G) The phosphorylation of TBK1 induced by ISD was enhanced in *Ppm1a* ^*–/—*^MEFs. Primary *Ppm1a* ^*+/+*^ and *Ppm1a* ^*–/—*^MEFs were left untreated or transfected with ISD (2 μg/ml) for the indicated times. The cell lysates were analyzed by immunoblotting with the indicated antibodies. (H) *Ppm1a* ^-/-^ MEFs were first infected with retrovirus expressing PPM1A-WT or PPM1A-R147G, after 48 h infection, cells were then transfected with (or without) ISD (3 μg/ml) for 7 h and followed by qRT-PCR analysis. *Ppm1a* ^*+/+*^ MEFs were used as positive control. The relative levels of *Ifnβ* mRNA in the control cells (*Ppm1a* ^*+/+*^, mock) was assigned a value of 1. (I) PPM1A deficiency suppressed the amplification of HSV-1. Primary *Ppm1a* ^*+/+*^ and *Ppm1a* ^*–/—*^MEFs were infected with HSV-1 at MOI of 5 for 16 h. The genomic DNA was extracted and the relative HSV genome copy numbers were measured with qRT-PCR analysis. The data in A–E, H and I are from one representative experiment of at least three independent experiments (means ± SD of triplicate assays in A-D and H-I, or duplicate in E). A two-tailed Student’s *t* test was used to analyze statistical significance. * P < 0.05; ** P < 0.01versus the control groups.

To better understand the biological importance of PPM1A in regulating antiviral signaling, we generated *Ppm*1a^–/—^mouse embryonic fibroblasts (MEFs) and examined the effects of *Ppm*1a deletion on the activation of STING signaling. We used a double-stranded DNA (dsDNA) containing 45 nucleotides, known as “IFN stimulatory DNA” (ISD), as a stimulator to activate the IFN-β signaling cascade in MEFs. As shown in Fig. 4A–D, the ISD-induced transcription of antiviral genes, including *Cxcl10*, *Ifnβ*, *Rantes*, and *Isg15*, were strongly increased in *Ppm*1a^*–/*—^MEFs compared to those in *Ppm*1a^*+/+*^ MEFs. In support of this, the level of IFN-β protein induced by ISD was also increased in *Ppm*1a^*–/*—^MEFs when measured with an enzyme-linked immunosorbent assay (Fig. 4E). Furthermore, levels of IRF3 dimerization and phosphorylation of endogenous TBK1 induced by ISD were also markedly increased in *Ppm*1a^*–/*—^MEFs, when compared to *Ppm*1a^*+/+*^ MEFs under the same experimental conditions (Fig. 4F-G). Additionally, we observed that the levels of *Ifnβ* at both mRNA and protein induced by HSV-1 virus infection were increased in *Ppm*1a^*–/—*^bone marrow-derived macrophages (BMDM), compared to the *Ppm*1a^*+/+*^ control (S3A and S3B Fig). We next sought to perform a rescue experiment to better understand the biological roles and the specificity of PPM1A in antiviral signaling using the *Ppm*1a^*–/*—^MEF cells. As shown in Figs. 4H and S4, expression of wild-type PPM1A, but not mutant PPM1A-R174G, could reverse the enhancement of *Ifnβ* expression induced by ISD treatment or HSV-1 infection in *Ppm*1a^*–/*—^MEFs. Taken together, these data indicated PPM1A play a negative role in STING-mediated antiviral signaling.

We next determined the biological function of PPM1A in regulating virus amplification. As shown in Fig. 4I, the copy numbers of HSV-1 genomic DNA were much lower in the *Ppm*1a^*–/*—^MEFs cells than in wild-type MEFs, suggesting that PPM1A is involved in regulating the amplification of DNA virus. Given that TBK1 also plays critical roles in anti-RNA-viral signaling, and that PPM1A suppresses TBK1 activity, we then sought to determine whether PPM1A also regulates the amplification of RNA virus. We measured the titer of the vesicular stomatitis mutant virus (VSVΔM51-GFP), a RNA virus which carries a single amino acid deletion (methionine 51) in the matrix (M) protein in VSV-GFP virus, in wild-type and *Ppm*1a^*–/*—^MEFs. As shown in S5A and S5B Fig, we observed much lower viral titers of VSVΔM51-GFP produced in *Ppm*1a^*–/*—^MEFs than in wild-type control cells. These results provide further biological evidence that PPM1A is a negative regulator of IRF3 activation, IFN-β production, and cellular antiviral response.

### TBK1 promotes STING aggregation

**Fig 5:**
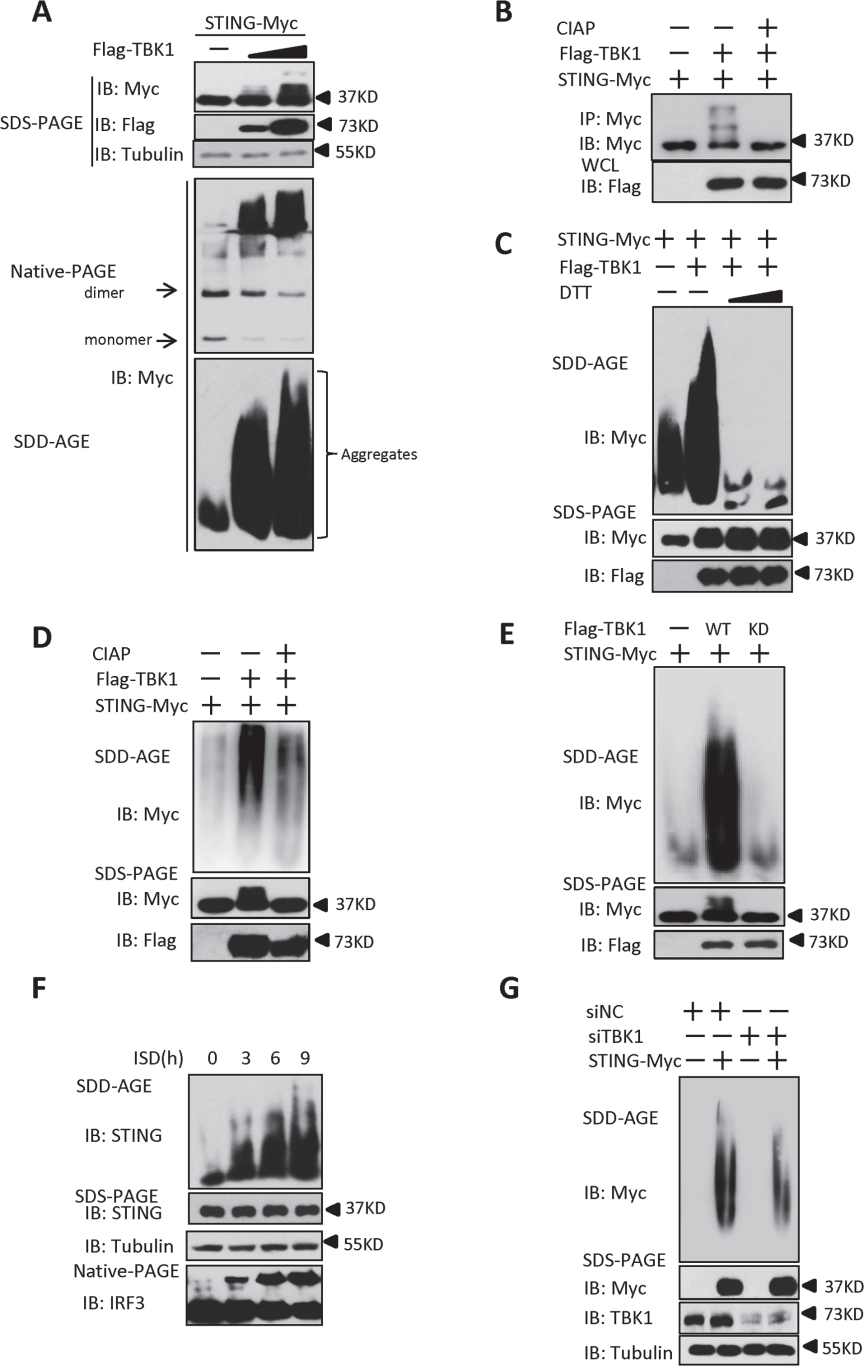
TBK1 promotes STING aggregation. (A) TBK1 promoted STING aggregation in a dose-dependent manner. HEK293 cells were transfected with STING-Myc expression plasmids (0.2 μg) together with the empty vector (2 μg) or increased dose of Flag–TBK1 expression plasmids (0.5 μg, 2 μg). At 30 h after transfection, the cell lysates were resolved by SDS-PAGE (top), native gel electrophoresis (middle), or SDD-AGE (bottom), and analyzed by immunoblotting with the indicated antibodies. (B) TBK1 induced STING phosphorylation. HEK293 cells were transfected with STING-Myc (0.5 μg) along with empty vector (2 μg) or Flag-TBK1 (2 μg), and 24 h later, cell lysates were immunoprecipitated with anti-Myc. The immunoprecipitates were treated with buffer or calf intestine phosphatase (CIAP) and analyzed by with SDS-PAGE, followed by immunoblotting. (C) DTT treatment reduced STING aggregation. HEK293 cells were transfected with STING-Myc expression plasmids together with the empty vector or Flag–TBK1 expression plasmid. At 30 h after transfection, the cell lysates were untreated or treated with 5 mM or 20 mM DTT for 30 min at room temperature, and then resolved by SDD-AGE (upper panel) or SDS-PAGE (lower panels), followed by immunoblotting analysis with the indicated antibodies. (D) CIAP treatment reduced the STING aggregation induced by TBK1. HEK293 cells were transfected with STING-Myc expression plasmids along with empty vector or Flag–TBK1 expression plasmids. At 24 h after transfection, the cells were lysed and then left untreated or treated with CIAP for 30 min at 37°C as indicated and analyzed with SDD-AGE and SDS-PAGE, followed by immunoblotting analysis. (E) TBK1 kinase activity was important for the induction of STING aggregation. HEK293 cells were transfected with the indicated plasmids for 24 h. The cell lysates were separated with SDD-AGE and SDS-PAGE, and analyzed by immunoblotting with the indicated antibodies. (F) Endogenous STING formed aggregates in response to stimulation with ISD in THP-1 cells. THP-1 cells were transfected with ISD (2 μg/ml) using Lipofectamine 2000 for the indicated times and lysed. The proteins were resolved with SDD-AGE and SDS-PAGE, and analyzed by immunoblotting with the indicated antibodies. (G) Knockdown of endogenous TBK1 attenuated STING aggregation in HEK293 cells. HEK293 cells were transfected with NC (40 nM) or a TBK1 (40 nM) RNAi oligonucleotide. At 48 h after transfection, the cells were transfected with vector encoding STING-Myc or empty vector for 24 h. The cell lysates were prepared and resolved with SDD-AGE (upper panel) and SDS-PAGE assays (lower panel), followed by immunoblotting with the indicated antibodies.

It has previously been reported that STING acts as a scaffold to assemble the protein complex containing TBK1 and IRF3, thus specifying and promoting the phosphorylation of IRF3 by TBK1 [25]. Our results showed that PPM1A plays a role in regulating the activation of both STING and TBK1 in a manner that depends on the catalytic activity of PPM1A, suggesting a potential regulatory relationship among these proteins. Because of the important role of STING in transducing antiviral signaling against DNA virus, we investigated whether and how the dynamic status of STING phosphorylation is regulated by TBK1 and PPM1A. To test whether TBK1 regulates STING, we transfected HEK293 cells with plasmids expressing TBK1 and STING, as shown in Fig. 5A (top panel), the expression of TBK1 caused band shifts of the STING on sodium dodecyl sulfate-polyacrylamide gel electrophoresis (SDS-PAGE) in a dose-dependent manner. The shifted bands appeared to be phosphorylated forms of STING, since calf intestinal alkaline phosphatase (CIAP) treatment abolished the band shifts (Fig. 5B), suggesting that TBK1 promotes STING phosphorylation. Interestingly, we noted that STING also formed high-molecular-weight products that stayed in the stacking gel on native PAGE (Fig. 5A, middle panel), when it was co-expressed with TBK1. These findings suggest that the TBK1-mediated phosphorylation of STING potentially promotes its polymerization.

Prion-like polymerization has recently been shown to be a conserved signal-transduction mechanism in both yeast and mammal [34,35]. Previous studies have suggested that in response to RNA viral infection, MAVS forms large prion-like aggregates that potently activate MAVS-mediated antiviral signaling [8]. A recent study [25] and our current study suggest that STING is potentially polymerized, and as demonstrated here, this polymerization of STING might be promoted directly by TBK1. Therefore, we examined whether STING aggregates behave like MAVS. To do so, we separated cell lysates with semi-denaturing detergent agarose gel electrophoresis (SDD-AGE), a method for detecting large protein polymers in studying prions [36]. As shown in S6A Fig, the overexpression of STING alone produced a smear of high-molecular weight aggregates at higher doses, albeit at a lower level. However, we found that levels of aggregation of STING were dramatically increased, when it was co-expressed with TBK1, suggesting that TBK1 promotes STING aggregation (Fig. 5A, bottom panel). Of note, STING aggregation required the N-terminal (amino acids 1–152) and C-terminal (amino acids 311–379) regions, since lacking either of these regions evidently affected the aggregation of STING (S6B and S6C Fig). Interestingly, we found that similar to MAVS [8], the aggregation of STING promoted by TBK1 was also very sensitive to Dithiothreitol (DTT) treatment (Fig. 5C), suggesting that STING aggregates are likely facilitated by the disulfide bond formation. Furthermore, we found that the aggregation of STING was also reduced when the lysates were treated with CIAP (Fig. 5D), revealing that the formation of STING aggregates is dependent on its phosphorylation. In a line with this, the kinase-dead form of TBK1 (TBK1-KD) failed to promote further STING aggregation (Fig. 5E). Taken together, these findings suggest that the TBK1-induced phosphorylation of STING promotes the aggregation of STING.

We next investigated whether STING aggregation occurs when THP-1 cells were stimulated with ISD. As shown in Fig. 5F, ISD treatment induced STING aggregation in a time-dependent manner. Consistently, similar results were obtained with HSV-1 infection (S6D Fig). Since overexpression of TBK1 promoted STING aggregation, we next tested whether TBK1 is necessary for the STING aggregation. As shown in an SDD-AGE assay, the knockdown of TBK1 appeared to attenuate STING aggregation in HEK293 cells (Fig. 5G). Collectively, these findings support the notion that TBK1 promotes STING aggregation, likely in a phosphorylation-dependent manner.

### Identification of specific phosphorylation residues in STING that contribute to its aggregation

**Fig 6:**
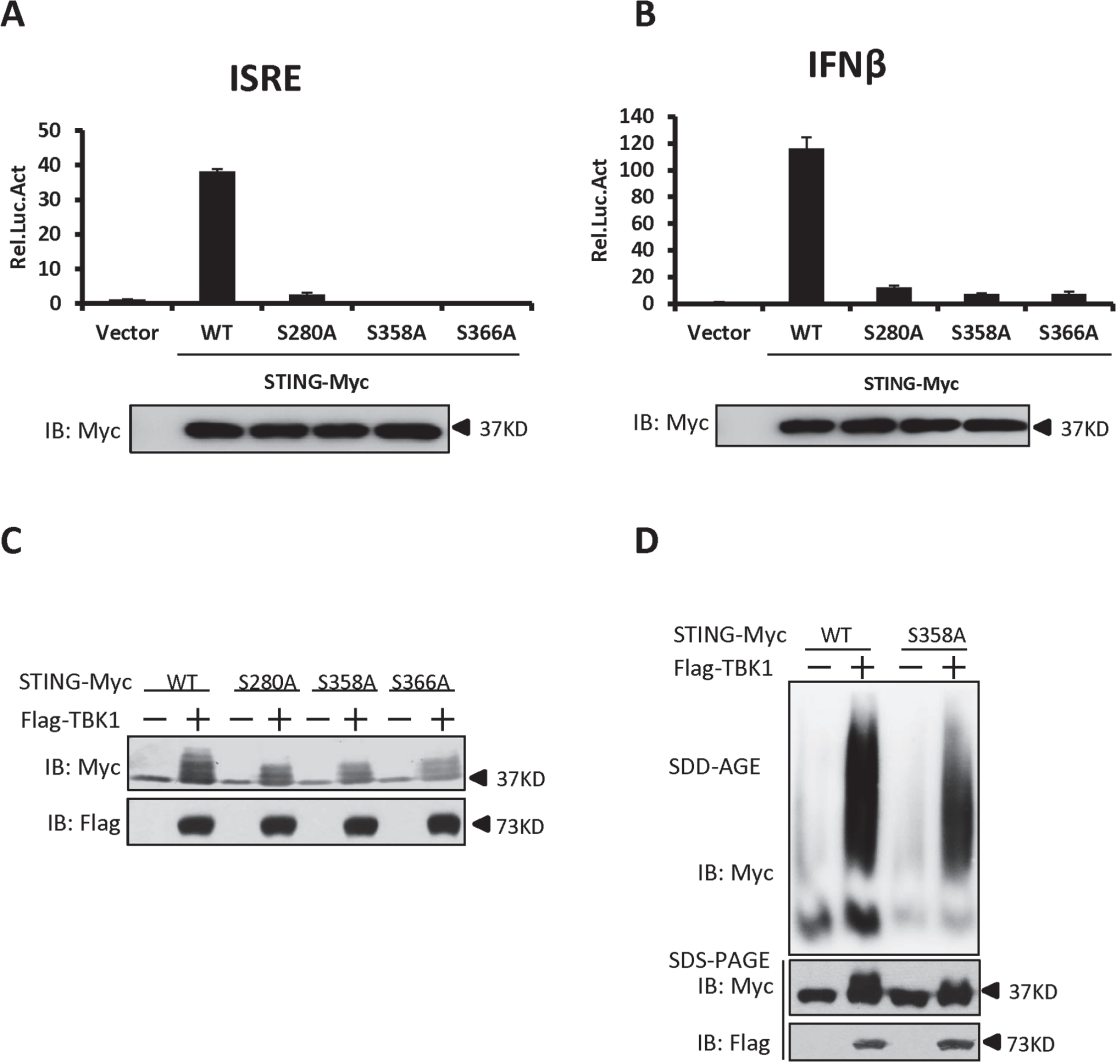
Identification of specific phosphorylation sites in STING that contribute to its aggregation. (A, B) The luciferase reporter assays showing activation of the ISRE (A) and IFN-β (B) promoter to different levels by that indicated STING Ser mutants. HEK293 cells were transfected with vectors encoding STING-WT or mutants as indicated. At 30 h after transfection, the cells were lysed for luciferase assays (upper panel) and immunoblotting assays (lower panel). (C) The phosphorylation of STING mediated by TBK1 was reduced in STING S280A, S358A, and S366A mutants. HEK293 cells were transfected with vectors encoding STING wild-type or its mutants, together with TBK1-encoding vector or empty vector, for 30 h. The cell lysates were analyzed by immunoblotting with the indicated antibodies. (D) The residue S358 of STING was important for its aggregation. HEK293 cells were transfected with vector encoding STING-WT or STING-S358A together with TBK1 expression vector or empty vector. At 30 h after transfection, the cell lysates were resolved by SDD-AGE (upper panel) or SDS-PAGE (lower panel) and analyzed by immunoblotting with the indicated antibodies. The data in A–B are from one representative experiment of at least three independent experiments (means ± SD of duplicate assays).

Having established that TBK1 promotes STING phosphorylation and aggregation, we then determined how TBK1-mediated STING phosphorylation and attempted to identify the putative phosphorylation residues in STING that are required for its activity and its aggregation as well. Based on the protein sequence of STING, we generated a library of human STING mutants in which serine (S) or threonine (T) residues were mutated to alanine (A) either individually, or two adjacent serine and/or threonine residues in combination, according to the method described previously [37]. Using this library, we performed luciferase reporter assays to test the potential roles of these serine and threonine residues in controlling STING function. As shown in luciferase reporter assays, mutation of S280, S358, or S366 to alanine remarkably reduced the activation of STING (Figs. 6A and B, and S7A-S7D), suggesting that these residues are critical for the STING activation to induce downstream signaling. Notably, further western blot analysis suggested that mutation of S280, S358, or S366 to alanine affected the TBK1-mediated phosphorylation of STING, since the shifted bands were apparently reduced (Fig. 6C). In addition to the above strategy, we also employed the HEK293 cells co-expressing TBK1 and STING to perform a mass spectrometric analysis and again identified the S358 site as the phosphorylated residue in STING (S8A–S8C Fig, S1 Text). We then asked whether the S358 residue in STING could be directly phosphorylated by TBK1 and performed an *in vitro* phosphorylation assay. As shown in S9 Fig, a stronger super-shift signal of wild-type STING induced by TBK1 could be detected, when compared to the STING-S358A mutant. Taken together, these findings support that S358 is one of the residues for phosphorylation targeted by TBK1 and the phosphorylation status of this site likely contributes to the full activation of STING.

We next tested whether the S358 residue contributes to the polymerization of STING by performing SDD-AGE assays. As shown in Fig. 6D, the S358A mutant of STING has a significant reduction in self-propagating polymer formation induced by TBK1, when compared to wild-type STING. These findings suggest that the S358 in STING is an important target residue phosphorylated by TBK1 and also contributes to the aggregation of STING.

### PPM1A directly dephosphorylates STING and prevents STING aggregation

**Fig 7:**
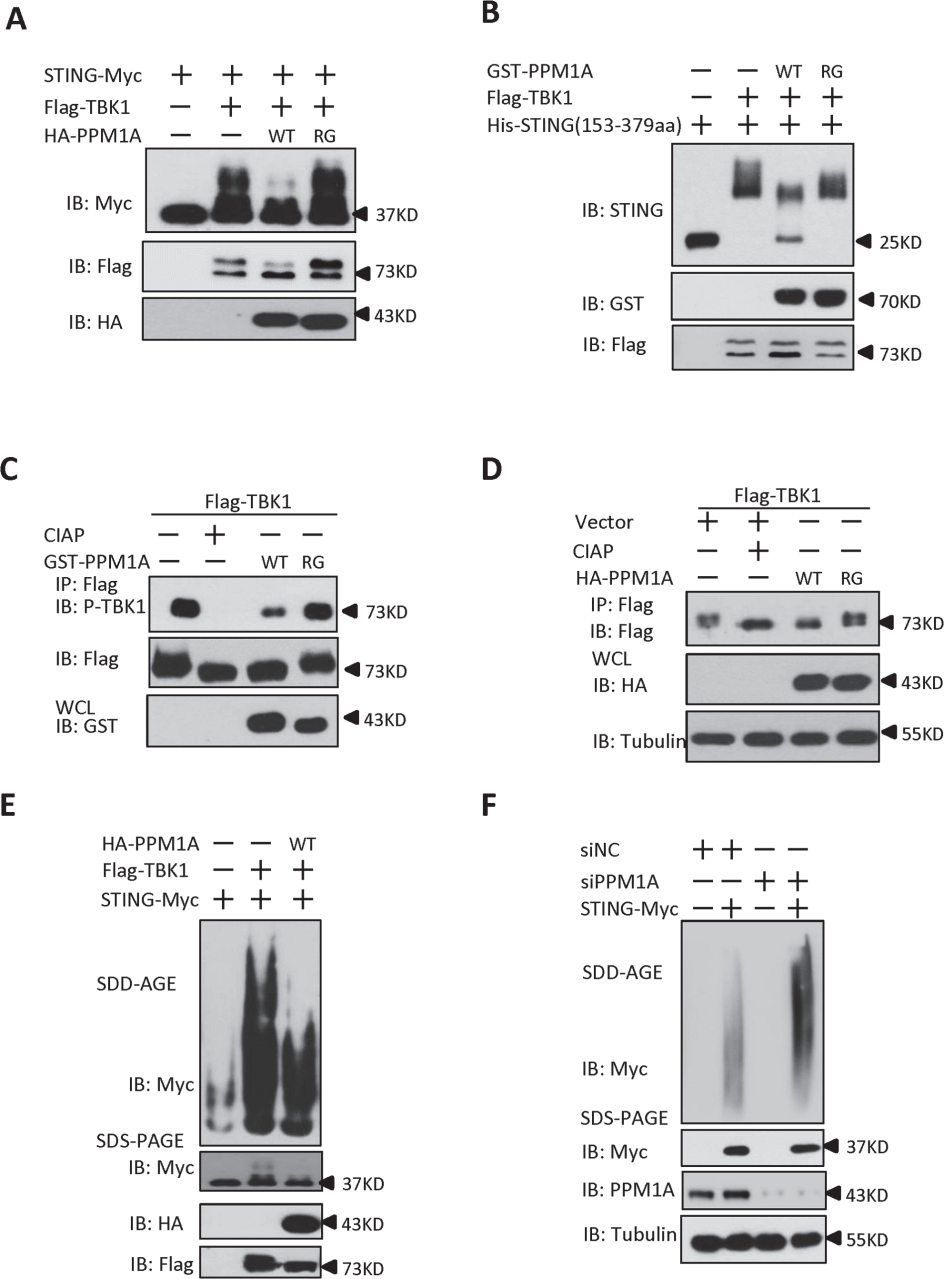
PPM1A directly dephosphorylates STING and TBK1 and prevents STING aggregation. (A) STING phosphorylation induced by TBK1 was inhibited by PPM1A-WT, but not PPM1A-R174G. HEK293 cells were transfected with indicated plasmids for 24 h, and cell lysates were separated by SDS-PAGE and analyzed by immunoblotting with antibodies. (B) STING was a direct target of PPM1A in an *in vitro* phosphatase assay. Purified His–STING (amino acids 153–379; 1 μg) and Flag–TBK1 (1 μg) were incubated in kinase buffer for 30 min at 30°C. After the samples were concentrated and the buffer was changed to phosphatase buffer, Purified GST–PPM1A-WT (1 μg) or GST-PPM1A-R174G protein (1 μg) was added, incubated for another 30 min at 30°C, and then analyzed by immunoblotting with the indicated antibodies. (C) TBK1 was a direct target of PPM1A in an *in vitro* phosphatase assay. Purified Flag–TBK1 (1 μg) and GST–PPM1A–WT or GST-PPM1A-R174G (1 μg) were incubated in phosphatase buffer for 30 min at 30°C and then analyzed by immunoblotting with the indicated antibodies. (D) PPM1A decreases phosphorylation of TBK1 in transfected HEK293 cells. HEK293 cells were transfected with Flag-TBK1 (0.5 μg) along with empty vector (2 μg) or HA-PPM1A-WT or HA-PPM1A-R174G (2 μg), and 24 h later, cell lysates were immunoprecipitated with anti-Flag beads. The immunoprecipitates were treated with buffer or CIAP as indicated and analyzed by immunoblotting with anti-Flag (upper panel). Expression of the transfected proteins was analyzed by immunoblotting with anti-HA (lower panel). (E) TBK1-induced STING aggregation was impaired by PPM1A. HEK293 cells were transfected with the indicated expression plasmids. At 30 h after transfection, the cell lysates were resolved with SDD-AGE (upper panel) or SDS-PAGE (lower panel) and analyzed by immunoblotting with the indicated antibodies. (F) PPM1A knockdown enhances STING aggregation in HEK293 cells. HEK293 cells were transfected with siNC (40 nM) or siPPM1A-2 (40 nM). At 48 h after transfection, the cells were transfected with empty vector or STING expression vector for 20 h. the cell lysates were resolved with SDD-AGE (upper panel) or SDS-PAGE (lower panel) and analyzed by immunoblotting with the indicated antibodies.

Having demonstrated that TBK1 plays an important role in controlling STING phosphorylation and aggregation, we next sought to ask whether PPM1A has an opposite effect in regulating STING phosphorylation and aggregation. Several lines of evidence suggest that PPM1A antagonizes TBK1 function and regulates STING phosphorylation and aggregation. First, PPM1A functions as a negative regulator of STING-induced antiviral signaling; second, PPM1A associates with both STING and TBK1; and third, PPM1A functions as a Ser/Thr phosphatase and its catalytic activity is required for regulating STING activation. To test this hypothesis, we first examined whether PPM1A affects the phosphorylation of STING induced by TBK1 in transfected cells. As shown in Fig. 7A, we found that PPM1A dramatically reduced the phosphorylation of STING in a phosphatase activity-dependent manner. Next we further determined whether STING is a direct target of PPM1A. We purified His–STING (amino acids 153–379), GST–PPM1A-WT and GST-PPM1A-R174G from bacteria, Flag–TBK1 from HEK293 cells, respectively, and performed *in vitro* phosphorylation and dephosphorylation assays. In these assays, His–STING (amino acids 153–379) was firstly used as the substrate to be phosphorylated by Flag–TBK1. Once the phosphorylation reaction was terminated, the reaction buffer was changed to phosphatase buffer and the GST–PPM1A-WT or GST-PPM1A-R174G was added for a subsequent phosphatase assay. As shown in Fig. 7B, GST–PPM1A-WT, but not GST-PPM1A-R174G, clearly reduced the levels of STING phosphorylation catalyzed by Flag-TBK1. To further test whether PPM1A target STING dephosphorylation through its S358 site, we used synthesized STING phosphopeptides corresponding to phospho-S358 as substrate, and performed an *in vitro* dephosphorylation assay, and found that GST-PPM1A-WT, but not GST-PPM1A-R174G, directly dephosphorylates pS358 peptide (S10A Fig). This finding provides direct biochemical evidence suggesting that PPM1A regulates de-phosphorylation of STING. Of note, we also found that the levels of TBK1 phosphorylation at Ser 172, were reduced by PPM1A both in the *in vitro* phosphatase assay (Fig. 7C) and in transfected cells (Fig. 7D), suggesting that TBK1 is also a direct target of PPM1A. By contrast, we found that PPM1A failed to dephosphorylate IRF3 at the Ser 396 residue, which is phosphorylated in response to TBK1 activation, under the same experimental conditions (S10B–S10C Fig).

Because PPM1A targets both TBK1 and STING, we tested whether the PPM1A-mediated dephosphorylation of STING and TBK1 affects STING aggregation. HEK293 cells were transfected with epitope-tagged STING and TBK1, with or without PPM1A. Cell lysates were subjected to SDD-AGE assays to examine STING aggregation. As shown in Fig. 7E, PPM1A evidently impaired TBK1-induced STING aggregation in the transfected HEK293 cells. Consistent with this, knockdown of PPM1A significantly enhanced the STING aggregation in HEK293 cells (Fig. 7F). Importantly, we consistently found the STING aggregation induced by ISD was significantly enhanced in *Ppm*1a^*–/*—^MEFs, compared to *Ppm*1a^*+/+*^ MEFs (S11 Fig)

Taken together, these findings suggest that TBK1-mediated STING phosphorylation is important for STING function and its self-propagating polymerization, whereas PPM1A prevents STING aggregation by directly dephosphorylating both STING and TBK1.

## Discussion

STING plays an important role in defending against DNA virus infection. Although the mechanism of STING activation has been studied extensively, much less is known about how the activation of STING is balanced to ensure the proper antiviral signal transduction, thereby avoiding deleterious effects on the host cells when responding to viral infection. In this study, we have demonstrated that PPM1A, a member of the PP2C family of Ser/Thr protein phosphatases, physically interacts with and dephosphorylates both STING and TBK1, thus negatively regulates antiviral signaling. Importantly, we found that whereas TBK1 promotes STING phosphorylation to induce a self-propagating polymerization, PPM1A antagonizes STING aggregation in a dephosphorylation-dependent manner. Thus, our study has established a novel mechanism by which PPM1A regulates antiviral signaling by antagonizing TBK1-mediated STING phosphorylation and aggregation, thereby maintaining innate immune homeostasis in the host cells.

### Dynamic phosphorylation/dephosphorylation balances the antiviral signaling induced by STING

The activation of protein kinases and the subsequent substrate phosphorylation are critical to antiviral signaling pathways [31,38–40]_.Viral infection or stimulation by dsDNA activates STING by relocating it from the ER to the Golgi and causing its assembly into punctate structures, where STING associates with TBK1 [41]. The association between STING and TBK1 promotes TBK1 activation, which further phosphorylates IRF3 and induces downstream target genes expression[25]. This demonstrates the importance of TBK1-mediated phosphorylation events, which are regulated by STING during antiviral signaling transduction. However, how STING is regulated by the TBK1 kinase remains unclear. In this study, we have presented evidence that STING is directly phosphorylated by TBK1 in a kinase activity-dependent manner. The mutation of putative phosphorylation sites in STING reduced the antiviral signaling of STING, suggesting that the phosphorylation of STING, mediated by TBK1, contributes to the full activation of STING. Therefore, STING and TBK1 reciprocally regulate each other to promote the rapid induction of antiviral signal transduction.

How is this feed-forward regulation between STING and TBK1 balanced? In this study, we have shown that PPM1A, a Ser/Thr phosphatase, physically interacts with both STING and TBK1. As shown in reporter assays, whereas ectopic expression of PPM1A significantly suppressed the STING-induced activation of IRF3 and the expression of the *IFNβ* gene, the knockdown of PPM1A markedly increased STING-mediated antiviral signaling (Figs. 2A, B and 3C, D). Consistent with this, *Ppm*1a^*–*/—^MEFs showed increased IRF3 dimerization and *Ifnβ* gene transcription after ISD stimulation. Particularly, *Ppm*1a^*–*/—^MEFs also exhibited reduced viral replication after HSV-1 or VSVΔM51-GFP virus infection, demonstrating its biological importance. Furthermore, our study suggests that PPM1A not only dephosphorylates TBK1, but also targets STING for dephosphorylation. Thus, the question is that, apart from TBK1, whether the PPM1A-mediated STING dephosphorylation also contributes to the IFNβ signaling transduction. In an effort to address this issue, we performed several experiments and obtained evidence supporting the important role of the PPM1A-mediated STING dephosphorylation in regulating the IFNβ signaling. First, PPM1A directly dephosphorylated STING, likely via its S358 site (Figs. 7A-B and S10A). Second and importantly, PPM1A could still reduce the STING phosphorylation induced by TBK1-S172E, a phosphorylation/ dephosphorylation-resistant form of TBK1, providing evidence that PPM1A directly dephosphorylated STING *in vivo* (S10D Fig). Third, the levels of *Ifnβ* expression induced by ISD in *Ppm*1a^*-/-*^ *MEF* cells ectopically expressing STING-S357A (corresponding to human STING-S358A mutant) were much lower than that in *Ppm*1a^-/-^ MEF cells expressing the wild-type STING (S12 Fig). Taken together, these findings support the notion that PPM1A functions as a phosphatase to balance antiviral signaling by dephosphorylating both STING and TBK1.

Of note, we observed that PPM1A fails to remove all the phosphorylation of STING/TBK1 in vitro or in vivo assays, opening an interesting possibility of the existence of other phosphatases that are involved in regulating STING/TBK1 dephosphorlyation. A previous study has reported that PPM1B influences the IFNβ signaling against RNA virus through dephosphorlyation of TBK1 [31], our study also showed knockdown of PPM1B increased the levels of IFNβ expression induced by HSV-1 infection (S13A–S13B Fig). Thus, PPM1B might also contribute to regulating STING signaling by controlling its phosphorylation status.

### PPM1A and TBK1 balance antiviral signaling by controlling STING aggregation

Two different mechanisms have been reported to explain the function of phosphorylation of STING in mediating the signaling transduction. Konno et al. previously reported that ULK1 phosphorylated STING at S366 to facilitate STING degradation and suppress IRF3 function [23]. However, a recent study provided evidence that STING phosphorylation at S366 by TBK1 is required for direct IRF3 recruitment and activation, but not for STING degradation [26]. In our study, we found that PPM1A dephosphorylated STING, but did not affect its degradation; since we didn’t observe that more stable STING proteins were present in *Ppm*1a^*-/-*^ MEF cells after ISD treatments, compared to wild-type control (S14 Fig).

Given the opposite roles of PPM1A and TBK1 in regulating the phosphorylation status of STING, and the fact that TBK1 promotes STING aggregation, we investigated the molecular mechanism underlying the action of PPM1A by examining whether PPM1A antagonizes STING aggregation by dephosphorylating it. We found that alteration of PPM1A levels by either overexpression or knockdown reduced or potentiated the aggregation of STING, respectively, suggesting an important role for PPM1A in the regulation of STING aggregation. We also found that the mutation of STING at residue S358 not only reduced the level of STING phosphorylation, but also reduced its aggregation, thus attenuating the antiviral activity of STING. Consistent with this, the kinase activity of TBK1 is necessary and sufficient for STING aggregation. These lines of evidence not only support the idea that STING aggregation is controlled by its phosphorylation status, but also argue that aggregation of STING on the ER or other membranes provide an ideal platform for the efficient downstream TBK1-mediated phosphorylation and activation of IRF3. This argument offers a better explanation of why the activation of TBK1 is not efficient to induce IRF3 activation in the absence of STING in response to cytosolic DNA.

Recent studies have suggested that both MAVS and ASC undergo a process of self-propagating polymerization upon induction by innate immune or inflammasome stimulators [34,35]. The present study suggests that STING also forms high-molecular-weight self-polymers, which is regulated by TBK1-mediated phosphorylation and PPM1A-mediated dephosphorylation. It will be interesting to investigate whether a phosphorylation/dephosphorylation balance also controls MAVS and ASC aggregation in the future.

## Materials and Methods

### Ethics statements

All animal studies were carried out in strict accordance with the recommendations in the Guide for the Care and Use of Laboratory Animals of the Ministry of Science and Technology of the People's Republic of China. The protocols for animal studies were approved by the Committee on the Ethics of Animal Experiments of the Institute of Zoology, Chinese Academy of Sciences (Approval number: IOZ15001).

### Cell culture and animals

HEK293, Hela, and Vero cells were obtained from Shanghai Cell Bank of Chinese Academy of Sciences, and maintained in Dulbecco’s modified Eagle’s medium (DMEM) supplemented with 10% fetal bovine serum, 1% penicillin, and 1% streptomycin. *Ppm*1a^+/+^ and *Ppm*1a^–/—^MEFs (the detailed information regarding this KO mouse has been described in the reference[42]) were generated from 13.5-day embryos and maintained in complete DMEM (described above) with 1 mM sodium pyruvate, 10 μM l-glutamine, 10 μM β-mercaptoethanol and 1% nonessential amino acids. THP-1 cells were maintained in RPMI 1640 medium supplemented with 10% fetal bovine serum, 1% penicillin, 1% streptomycin, 10 μM β- mercaptoethanol, and 5 mM HEPES. Bone marrow-derived macrophages were prepared as previously described with modification [43]. Briefly, bone marrow cells were harvested from femurs and tibiae of mice, and cultured in complete DMEM containing conditioned media from L929 cell culture for five days, and then cells are collected for experiments.

### Antibodies

Rabbit anti-phospho-TBK1, anti-phospho-IRF3 and anti-TBK1 antibodies were from Cell Signaling Technology; rabbit anti-Tubulin and anti-IRF3 antibodies were from Santa Cruz Biotechnology; mouse anti-GAPDH antibody was from Sungene Biotechnology; mouse anti-PPM1A antibody was from Abcam; mouse anti-Flag and rabbit anti-HA antibodies were from Sigma; rabbit anti-Myc antibody was from MBL. Mouse or rabbit anti-STING antiserum was raised against recombinant human STING (amino acids 221–379). Mouse polyclonal antibody recognizing mouse STING was kindly provided by Dr. Zhengfan Jiang (Peking University) [44]

### Plasmids

Mammalian expression plasmids encoding Myc-tagged STING, hemagglutinin (HA)-tagged PPM1A, Flag-tagged TBK1, HA-tagged TBK1 and Flag-tagged IRF3 were constructed with standard molecular biology techniques. Various mutants, including STING mutants, PPM1A-R174G, Flag-TBK1-KD (K38A), Flag-IRF3-5D (S396D, S398D, S402D, T404D, S405D), were generated by PCR using *Pfu* Turbo DNA polymerase. The IFNβ-Luc and ISRE-Luc have been described previously [45].

### Transfection and luciferase reporter analysis

HEK293 cells were seeded in 24-well plates at a density of 1.0 × 10^5^ cells per well and transfected on the following day with the standard calcium phosphate transfection method. A pRSV/LacZ reporter plasmid (50 ng) and a firefly luciferase reporter plasmid (50 ng) were transfected together with the indicated expression plasmids. In the same experiment, empty control plasmid was added to ensure that the same amount of total DNA was transfected. Luciferase activity was measured at the indicated time point, and normalized to the LacZ activity. All reporter assays were repeated at least three times.

### RNA interference (RNAi) and lentiviral infection

HEK293 cells were transfected with siRNA at a final concentration of 40 nM with the standard calcium phosphate transfection method. After 48 h, the cells were transfected with the indicated plasmids using Lipofectamine 2000 (Invitrogen) for 20 h and then harvested for analysis. THP-1 cells were infected with a pLL3.7 lentiviral vector carrying a target PPM1A sequence or the non-targeting sequence (shRNA-NC) for 72 h, and then cells were untreated or infected with HSV-1 for 6 h. The knockdown efficiency was determined with a qRT-PCR analysis. The siRNA sequences were as follows (only the sense strand is shown): NC (non-targeting), TTCTCCGAACGTGTCACGT; PPM1A-1, GAGGAATGTTATTGAAGCC; PPM1A-2, GTACCTGGAATGCAGAGTA; TBK1, TCAAGAACTTATCTACGAA. The sequences for shRNA PPM1A were the same as siPPM1A-1 and siPPM1A-2. The sequences for shRNA PPM1B were as follows: PPM1B-1: GGGAAAAGGAGCGAATCCA; PPM1B-2: GCTCTGTGAATATGTTAAA.

### Co-immunoprecipitation, immunoblotting analysis, confocal microscopy, native PAGE, virus plaque assay and ELISA assay

These experiments were performed as previously described [45]. Briefly, for virus plaque assays, confluent Vero cells were used for infection with the diluted virus of VSVΔM51-GFP for 1 hour. Culture medium containing 2% methylcellulose was overlaid and incubated for about 36 h. Cells were then fixed for 15 min with methanol and further stained with 1% crystal violet to display plaques. Plaques were counted to determine the viral titer as plaque-forming units per ml.

### SDD-AGE assay

The cells were transfected as indicated and then washed with phosphate-buffered saline (PBS), the cells were resuspended with lysis buffer (0.5% Triton X-100, 50mM Tris-HCl, 150mM NaCl, 10% Glycerol,). The supernatants were separated by 1.5% SDD-AGE as previously described [45].

### Measurement of HSV-1 genomic DNA copy numbers

MEFs were plated in six-well plates and grown to 80% confluence overnight. The cells were washed with PBS and infected with HSV at 5 MOI at 37°C in serum-free DMEM for 1 h. The cells were then washed with warm PBS, cultured in complete DMEM for 16 h, and their genomic DNA was extracted. The HSV-1 genomic DNA copy numbers were determined with real-time qRT-PCR with HSV-1-specific primers with the following sequence: 5′-TGGGACACATGCCTTCTTGG-3′ and 5′-ACCCTTAGTCAGACTCTGTTACTTACCC-3′.

### Purification of GST- and His-tagged recombinant proteins

GST-PPM1A was cloned into the pGEX-4T-1 vector, and construct encoding His-tagged STING (amino acids 153–379) was cloned into the pET-28a vector. The fusion proteins were purified from the cell lysates using glutathione–Sepharose beads (Sigma) or Ni–Sepharose beads (GE), according to protocols of the manufacturers. The protein concentrations were measured with a Bradford Protein Assay (Bio-Rad).

### *In vitro* pull-down

Purified STING (amino acids 153–379; 1 μg) and purified PPM1A (1 μg) or GST (1 μg) were incubated with glutathione–Sepharose beads at 4°C overnight with rotation. The glutathione–Sepharose beads were washed three times with washing buffer (50 mM Tris-HCl, pH7.5, 200 mM NaCl, 10% glycerol, 0.1% Triton X-100) for 5 min at 4°C with rotation. The association between STING and PPM1A was assayed by immunoblotting.

### Phosphatase assays *in vitro*

HEK293 cells were transfected with the Flag–TBK1 expression plasmid using the standard calcium phosphate transfection method. The Flag–TBK1 protein was immunoprecipitated from cell extracts with anti-Flag beads. After the immunoprecipitated Flag–TBK1 was washed three times with washing buffer, and incubated with the Flag peptide at 4°C for 30 min with rotation for three times. The collected supernatants were concentrated with centrifugal filter units (Millipore) and the protein concentrations were measured with a Bradford protein assay (Bio-Rad). Flag–TBK1 (1 μg) and purified STING (amino acids 153–379; 1 μg) were incubated in kinase buffer (25 mM Tris-HCl, pH 7.5, 100 mM NaCl, 5 mM MgCl_2_, 5 mM ATP, 1 mM DTT) for 30 min at 30°C. The proteins were then left untreated or concentrated with centrifugal filter units and exchanged with phosphatase buffer (250 mM imidazole, 1 mM EGTA, 2 mM DTT, 25 mM MgCl_2_, 0.1% BSA). Purified GST-PPM1A-WT or GST-PPM1A-R174G (1 μg) was added and incubated for 30 min at 30°C. The phosphatase reaction was then terminated by boiling it in protein sample buffer and followed with an immunoblotting analysis.

### Malachite green phosphatase assay

The sequence of synthetic phosphopeptide was as follow: STING^p358S^(STSTM**pS**QEPEL). The phosphorylated amino acid is indicated in bold. The peptide was synthesized by Sangon Biotech, phosphopeptide phosphatase assay was performed with malachite green phosphatase assay kit (BioAssay Systems, POMG-048) according to the manufacturer’s instructions.

### Yeast two-hybrid screening

To perform the yeast two-hybrid screening, we cloned STING (amino acids 153–379) into the bait vector. A human spleen DNA library (Agilent) was screened according to the recommendations of the manufacturer.

### Quantitative RT-PCR

Total RNA was extracted using TRIZOL reagent (Invitrogen). cDNA was synthesized using SuperScript III First-Strand cDNA Synthesis kit (Invitrogen). Real-time PCR was performed using SYBR Green Master Mix (Thermo) in triplicate on a Bio-Rad iCycler iQ5 PCR Thermal Cycler. Relative levels of mRNA were normalized to the GAPDH RNA levels in each sample. 2^-ΔΔCt^ method was used to calculate relative expression changes. Data shown are the relative abundance of the mRNA as indicated to that of control groups. The primers used were as follows (5′–3′):

hGAPDH-S: ATGACATCAAGAAGGTGGTG

hGAPDH-AS: CATACCAGGAAATGAGCTTG

hIFN-β-S: AGGACAGGATGAACTTTGAC

hIFN-β-AS: TGATAGACATTAGCCAGGAG

hPPM1A-S: AGGGGCAGGGTAATGGGTT

hPPM1A-AS: GATCACAGCCGTATGTGCATC

mGAPDH-S: AACTTTGGCATTGTGGAAGG

mGAPDH-AS: ACACATTGGGGGTAGGAACA

mCxcl-10-S: GAATCCGGAATCTAAGACCATCAA

mCxcl-10-AS: GTGCGTGGCTTCACTCCAGT

mIFN-β-S: ATGGTGGTCCGAGCAGAGAT

mIFN-β-AS: CCACCACTCATTCTGAGGCA

mISG15-S: GGTGTCCGTGACTAACTCCAT

mISG15-AS: TGGAAAGGGTAAGACCGTCCT

mRANTES-S: CTCACCATATGGCTCGGACA

mRANTES-AS: ACAAACACGACTGCAAGATTGG

### Statistical analyses

All statistical analyses are shown as means ± SD. Significant differences between values under different experimental conditions were carried out using two-tailed Student’s t-test. For all tests, a p value of less than 0.05 was considered statistically significant.

### Accession numbers

The mRNA sequence data for genes described in this study can be found in the NCBI under the following accession numbers: *Homo sapiens* PPM1A (NM_021003.4), *Homo sapiens* STING(NM_198282.3), *Mus musculus* STING (NM_028261.1), *Homo sapiens* TBK1 (NM_013254.3), *Homo sapiens* IRF3 (NM_001571.5).

## Supporting Information

S1 Text Supplemental experimental procedures. (DOCX)

S1 Fig STING interacts with PPM1A. (A) Detection of the interaction between STING and PPM1A in the yeast two-hybrid system. The competent yeast cdc25H strain was transformed with the plasmids as indicated. pSos-MAFB and pMyr-MAFB were used as a positive control, and pSos-MAFB and pMyr-Lamin C were used as a negative control. (B) STING interacted with PPM1A in an *in vitro* protein-binding assay. Purified His–STING (amino acids 153–379; 1 μg) was incubated with purified GST–PPM1A (1 μg) or GST control protein (1 μg), and then pulled down with Ni–Sepharose beads, followed by immunoblotting analysis with the indicated antibodies.
(PDF)

S2 Fig Knockdown of PPM1A has no effect on the IRF3-5D-induced activation of ISRE. (A)Knockdown of PPM1A had no effect on the IRF3-5D-induced activation of ISRE. HEK293 cells were transfected with siNC or siPPM1A-2 (40 nM). Forty-eight hours after transfection, the cells were transfected with empty vector or IRF3-5D together with reporter constructs for 20 h using Lipofectamine 2000. The cells were lysed for luciferase assays (upper panel) and immunoblotting assays (lower panel). (B)The *Ppm1a* knockdown efficiency by the shRNA lentivirus was measured in THP-1 cells. THP-1 cells were infected with a lentivirus targeting PPM1A or NC for 72 h and then cells were lysed and total RNA were isolated for qRT-PCR analysis. Relative levels of mRNA were normalized to the GAPDH RNA levels in each sample. The Ppm1a mRNA abundance of the control group (shRNA-NC) was assigned a value of 100. The data in A and B is from one representative experiment of three independent experiments (means ± SD of duplicate assays in A or triplicate assays in B). A two-tailed Student’s t test was used to analyze statistical significance, n.s., No Significance, versus control groups.
(PDF)

S3 Fig Enhanced antiviral signaling in *Ppm1a* ^*-/-*^ BMDM cells. (A)The level of *Ifnβ* mRNA was increased in *Ppm1a* ^*–/—*^BMDMs post HSV-1 infection. *Ppm1a* ^*+/+*^ and *Ppm1a* ^–/—^BMDMs were left uninfected or infected with HSV-1(10 MOI) for 6 h, followed by qRT-PCR analysis. Relative levels of mRNA were normalized to the GAPDH RNA levels in each sample. Data shown are the relative abundance of *Ifnβ* to control groups. (B) The production of IFNβ protein was enhanced in *Ppm1a* ^-/-^ BMDMs post HSV-1 infection. *Ppm1a* ^+/+^ and *Ppm1a* ^-/-^ BMDMs were left uninfected or infected with HSV-1(10 MOI) for 24 h, and then the supernatants were collected for measurement of IFNβ protein by ELISA. The data in A-B is from one representative experiment of three independent experiments (means ± SD of triplicate assays in A or duplicate in B). A two-tailed Student’s *t* test was used to analyze statistical significance, * P < 0.05 versus the control groups.
(PDF)

S4 Fig Expression of PPM1A reverses the enhancement of *Ifnβ* expression induced by HSV-1 infection in *Ppm1a* ^*–/—*^MEFs. *Ppm1a* ^*-/-*^ MEFs were first infected with lentivirus expressing PPM1A-WT or PPM1A-R147G, after 48 h infection, cells were then infected with (or without) 1 MOI of HSV-1 for 6 h and followed by qRT-PCR analysis. *Ppm1a* ^*+/+*^ MEFs were used as positive control. The relative level of Ifnβ mRNA in the control cells (*Ppm1a* ^*+/+*^, mock) was assigned a value of 1.
(PDF)

S5 Fig PPM1A deficiency suppresses VSVΔM51-GFP amplification. (A-B) Primary *Ppm1a* ^*+/+*^ and *Ppm1a* ^*-/-*^ MEFs were infected with 0.1 MOI of VSVΔM51-GFP virus for 24 h, and then cells were imaged by fluorescence microscopy (A) and the supernatants were collected and virus titer was measured by plaque assay (B).The data in B is from one representative experiment of three independent experiments (means ± SD of duplicate assays). A two-tailed Student’s t test was used to analyze statistical significance, * P < 0.05 versus the control groups.
(PDF)

S6 Fig STING forms aggregates. (A) STING formed high-molecular-weight aggregates in a dose-dependent manner. HEK293 cells were transfected with increased dose of STING-Myc (0.1 μg, 0.5 μg, 1 μg), and 24 h later, the cell lysates were resolved with SDD-AGE (upper panel) or SDS-PAGE (lower panel) and analyzed by immunoblotting with the indicated antibodies. (B) Only the full length of STING could form aggregates. HEK293 cells were transfected with STING-Myc (amino acids 1–379), STING-Myc (amino acids 1–310) or STING-Myc (amino acids 153–379), 24 h later, cells were lysed and analyzed by immunoblotting with the indicated antibodies. (C) Both the N- and C-terminal of STING are important for its aggregates induced by TBK1. HEK293 cells were transfected with STING-Myc (amino acids 1–379), STING-Myc (amino acids 1–310) or STING- Myc (amino acids 153–379) together with empty vector or Flag-TBK1, 24 h later, the cell lysates were resolved with SDD-AGE (upper panel) or SDS-PAGE (lower panel) and analyzed by immunoblotting with the indicated antibodies. (D) THP-1 cells were infected with 1 MOI of HSV-1 as indicated times and cells were lysed for SDD-AGE or SDS-PAGE assay.
(PDF)

S7 Fig Identification of specific serine or threonine residues in STING for STING activation. (A-D) Identification of STING Ser/Thr residues which are required for the activation of ISRE (A, B) and IFNβ (C, D) promoters. HEK293 cells were transfected with vectors encoding the indicated STING WT and mutants. At 30 h after transfection, the cells were lysed for luciferase assays (upper panel) and immunoblotting assays (lower panel).
(PDF)

S8 Fig Identification of phosphorylated residues in STING by the mass spectrometric analysis. (A)The localizations of identified phosphorylation sites by mass spectrometry are shown, together with the structure of STING, in which cytoplasmic, membrane and non-cytoplasmic regions of STING were annotated by InterProScan (http://www.ebi.ac.uk/interpro/search/sequence-search). The blue rectangle represents non-cytoplasmic domain, the red rectangle represents transmembrane region, and the green rectangle represents cytoplasmic domain. (B)The representative identified fragment ions, including b and y ions, from MS/MS spectra for S358 site of STING. “ph” means phosphorylation. Specific y ions surrounding phosphorylated S358 were identified, indicating a reliable identification. (C)Sequence alignment for STING protein sequences showed evolutionary conservation of S358 among mammals.
(PDF)

S9 Fig The residue S358 of STING is a direct target site of TBK1. Purified His-STING-WT (amino acids 153–379,1 μg) or its mutant STING-S358A(1 μg) and Flag-TBK1 (1 μg) were incubated in kinase buffer for 30 min at 30°C and then analyzed by immunoblotting with the indicated antibodies.
(PDF)

S10 Fig PPM1A directly dephosphorylates STING but not IRF3. (A) Phosphopeptide assay was analyzed by Malachite phosphatase green assay. Synthesized STING phosphopeptide corresponding to phospho-S358 peptide was used as substrate, and incubated with equal amounts of GST, GST-tagged PPM1A-WT or PPM1A-R174G. The reaction was carried out for 60 minutes at 30°C and free phosphate was measured by malachite green assay. (B-C)TBK1, but not IRF3, was a direct target of PPM1A in an in vitro phosphatase assay. HEK293 cells were first transfected TBK1 alone (C) or in combination with IRF3 (B), respectively, and then lysed for co-IP experiments to purify the phosphorylated TBK1 and IRF3 proteins by the Flag peptides. Purified phosphorylated Flag-IRF3 (1 μg) (B) or Flag-TBK1(1 μg) (C) with the increased dose (0.5 μg, 1 μg) of GST-PPM1A-WT or GST-PPM1A-R174G were incubated in phosphatase buffer for 30 min at 30°C and then analyzed by immunoblotting with the indicated antibodies. (D) PPM1A dephosphorylated TBK1-WT- or TBK1-S172E-induced STING phosphorylation. HEK293 cells were transfected with plasmids as indicated, 24 h later, the cell lysates were resolved with SDS-PAGE and analyzed by immunoblotting with the indicated antibodies. The data in A is from one representative experiment of three independent experiments (means ± SD of triplicate assays).
(PDF)

S11 Fig STING aggregation was increased in *Ppm1a* ^-/-^ MEF cells after ISD treatment. *Ppm1a* ^+/+^ and *Ppm1a* ^-/-^ MEFs were first infected with lentivirus expressing STING-Myc, 36 h post-infection, cells were transfected with or without ISD(2 μg/ml) for 6 h, and then lysed for SDD-AGE or SDS-PAGE assay.
(PDF)

S12 Fig The S357 site in mouse STING is targeted by PPM1A. *Ppm1a* ^*+/+*^ and *Ppm1a* ^*-/-*^ MEFs were first infected with retrovirus expressing STING-WT, STING-S357A or empty vector, at 48 h post-infection, cells were then transfected with ISD(3 μg/ml) for 7 h and followed by qRT-PCR analysis. Data shown are the relative abundance of *Ifnβ* to control groups. The data is from one representative experiment of three independent experiments (means ± SD of triplicate assays). A two-tailed Student’s t test was used to analyze statistical significance. n.s., No Significance; *** P < 0.001 versus the control groups.
(PDF)

S13 Fig Knockdown of PPM1B increases the expression of *IFNβ* induced by HSV-1 infection in THP-1 cells. (A-B) THP-1 cells were first infected with a lentivirus targeting PPM1B for knockdown or NC for 72 h and then cells were infected with 1 MOI of HSV-1for 6 h and total RNA were isolated for qRT-PCR analysis. Relative mRNA levels of *IFNβ* (A) and PPM1B (B) were normalized to the GAPDH RNA levels in each sample. The *IFNβ* mRNA abundance of the control group (shRNA-NC, mock) was assigned a value of 1. The PPM1B mRNA abundance of the control group (shRNA-NC) was assigned a value of 100. The data in A and B is from one representative experiment of three independent experiments (means ± SD of triplicate assays in A and B). A two-tailed Student’s t test was used to analyze statistical significance, * P < 0.05, versus control groups.
(PDF)

S14 Fig PPM1A has no apparent role in influencing the protein turnover of STING. *Ppm1a* ^+/+^ and *Ppm1a* ^-/-^MEF cells were transfected with ISD (4 μg/ml) as indicated times and cells were collected and lysed for SDS-PAGE assay.
(PDF)
