# Supplementary material for: PPM1A Regulates Antiviral Signaling by Antagonizing TBK1-Mediated STING Phosphorylation and Aggregation
Source: PLoS Pathog. 2015 Mar 27;11(3):e1004783. doi: 10.1371/journal.ppat.1004783 (PMC4376777; doi:10.1371/journal.ppat.1004783)
Supplement: S1 Text — (DOCX) [file ppat.1004783.s001.docx]

**Supplemental Experimental Procedures**

**Phosphorylation site identification by mass spectrometry**

HEK293 cells were transfected with STING-Myc along with empty vector or Flag-TBK1, and 24 h later, cell lysates were immunoprecipitated with anti-Myc beads. The purified STING protein was separated by SDS-PAGE. The bands corresponding to STING and phosphorylated STING were cut into small pieces, and was in-gel digested by trypsin using the method previously described [1]. The digested peptides were desalted using C18-StageTips (Thermo Fisher Scientific, San Jose, CA), and the eluted peptides were analyzed on an LTQ Orbitrap Velos (Thermo Fisher Scientific) using a MS survey scan followed by 20 MS/MS by collision-induced dissociation (CID) [2]. Raw Data was processed using MaxQuant (version: 1.3.0.5) against human protein sequence database from UniProt according to the published procedure with some modifications [2]. The modification of phospho (STY) instead of acetyl (K) was used as a variable modification, otherwise the same. Only phosphorylation sites with a false discovery rate (FDR) less than 1% were considered.

**References**

1. Guo X, Shen J, Xia Z, Zhang R, Zhang P, et al. (2010) Proteomic analysis of proteins involved in spermiogenesis in mouse. J Proteome Res 9: 1246-1256.

2. Sun G, Jiang M, Zhou T, Guo Y, Cui Y, et al. (2014) Insights into the lysine acetylproteome of human sperm. J Proteomics 109C: 199-211.
