# Supplementary figures and images for: PPM1A Regulates Antiviral Signaling by Antagonizing TBK1-Mediated STING Phosphorylation and Aggregation
Source: PLoS Pathog. 2015 Mar 27;11(3):e1004783. doi: 10.1371/journal.ppat.1004783 (PMC4376777; doi:10.1371/journal.ppat.1004783)

**A**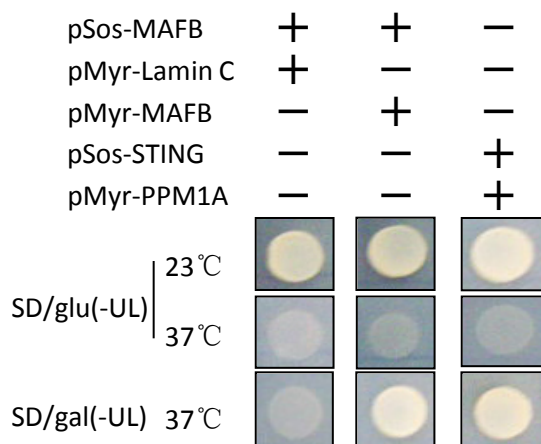**B**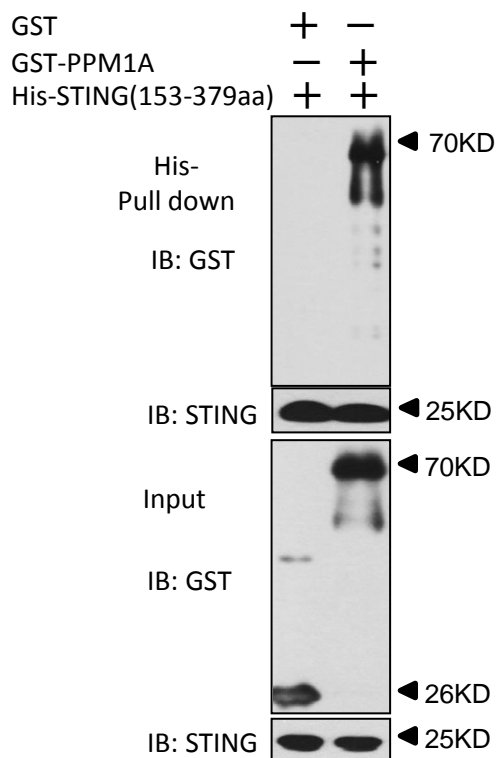

Supplement: S1 Fig — (A) Detection of the interaction between STING and PPM1A in the yeast two-hybrid system. The competent yeast cdc25H strain was transformed with the plasmids as indicated. pSos-MAFB and pMyr-MAFB were used as a positive control, and pSos-MAFB and pMyr-Lamin C were used as a negative control. (B) STING interacted with PPM1A in an in vitro protein-binding assay. Purified His–STING (amino acids 153–379; 1 μg) was incubated with purified GST–PPM1A (1 μg) or GST control protein (1 μg), and then pulled down with Ni–Sepharose beads, followed by immunoblotting analysis with the indicated antibodies. (PDF) [file ppat.1004783.s002.pdf]

**A**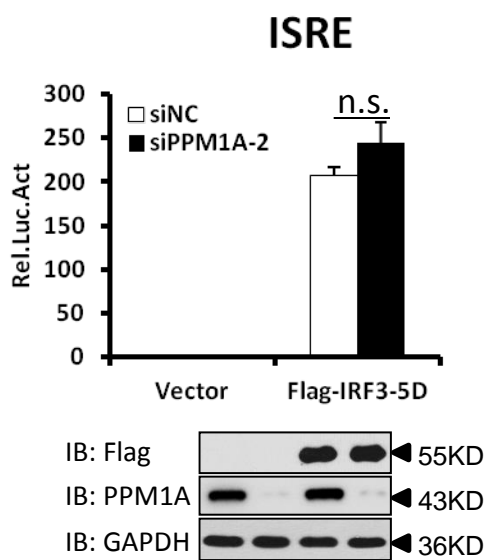**B**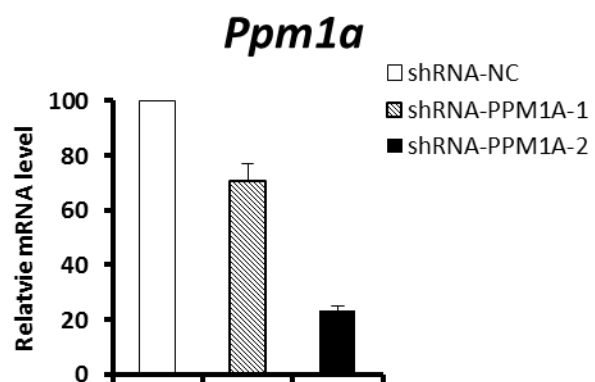

Supplement: S2 Fig — (A)Knockdown of PPM1A had no effect on the IRF3-5D-induced activation of ISRE. HEK293 cells were transfected with siNC or siPPM1A-2 (40 nM). Forty-eight hours after transfection, the cells were transfected with empty vector or IRF3-5D together with reporter constructs for 20 h using Lipofectamine 2000. The cells were lysed for luciferase assays (upper panel) and immunoblotting assays (lower panel). (B)The Ppm1a knockdown efficiency by the shRNA lentivirus was measured in THP-1 cells. THP-1 cells were infected with a lentivirus targeting PPM1A or NC for 72 h and then cells were lysed and total RNA were isolated for qRT-PCR analysis. Relative levels of mRNA were normalized to the GAPDH RNA levels in each sample. The Ppm1a mRNA abundance of the control group (shRNA-NC) was assigned a value of 100. The data in A and B is from one representative experiment of three independent experiments (means ± SD of duplicate assays in A or triplicate assays in B). A two-tailed Student’s t test was used to analyze statistical significance, n.s., No Significance, versus control groups. (PDF) [file ppat.1004783.s003.pdf]

**A**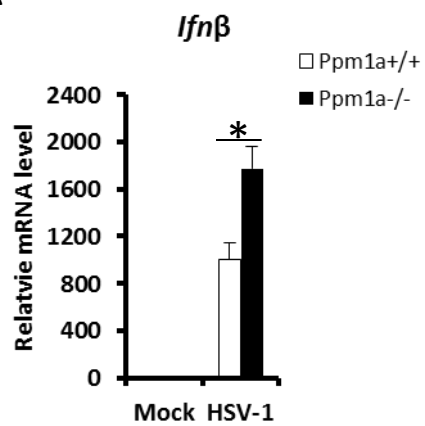**B**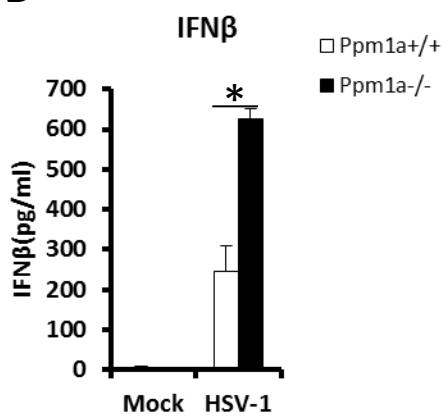

Supplement: S3 Fig — (A)The level of Ifnβ mRNA was increased in Ppm1a –/—BMDMs post HSV-1 infection. Ppm1a +/+ and Ppm1a –/—BMDMs were left uninfected or infected with HSV-1(10 MOI) for 6 h, followed by qRT-PCR analysis. Relative levels of mRNA were normalized to the GAPDH RNA levels in each sample. Data shown are the relative abundance of Ifnβ to control groups. (B) The production of IFNβ protein was enhanced in Ppm1a -/- BMDMs post HSV-1 infection. Ppm1a +/+ and Ppm1a -/- BMDMs were left uninfected or infected with HSV-1(10 MOI) for 24 h, and then the supernatants were collected for measurement of IFNβ protein by ELISA. The data in A-B is from one representative experiment of three independent experiments (means ± SD of triplicate assays in A or duplicate in B). A two-tailed Student’s t test was used to analyze statistical significance, * P < 0.05 versus the control groups. (PDF) [file ppat.1004783.s004.pdf]

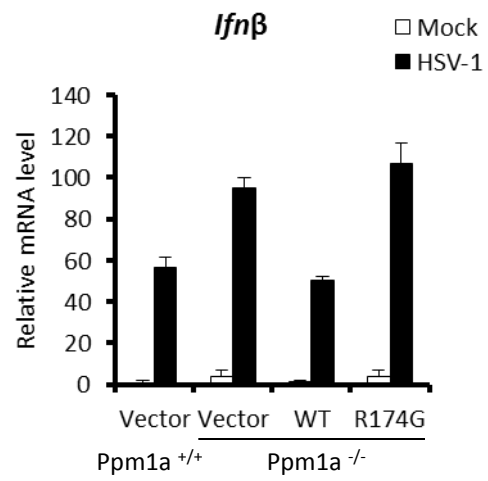

Supplement: S4 Fig — Ppm1a -/- MEFs were first infected with lentivirus expressing PPM1A-WT or PPM1A-R147G, after 48 h infection, cells were then infected with (or without) 1 MOI of HSV-1 for 6 h and followed by qRT-PCR analysis. Ppm1a +/+ MEFs were used as positive control. The relative level of Ifnβ mRNA in the control cells (Ppm1a +/+, mock) was assigned a value of 1. (PDF) [file ppat.1004783.s005.pdf]

**A**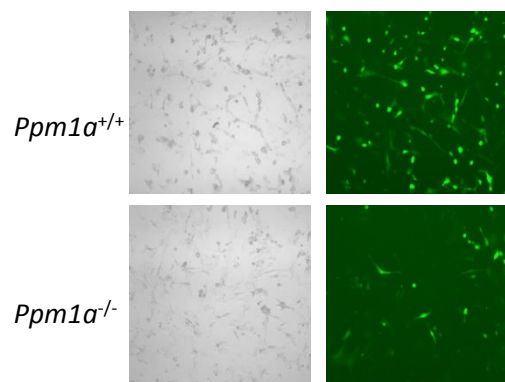**B**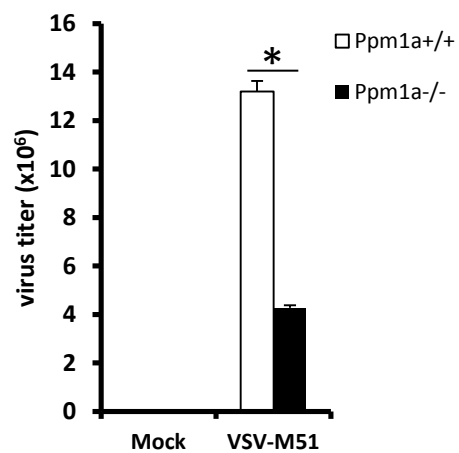

Supplement: S5 Fig — (A-B) Primary Ppm1a +/+ and Ppm1a -/- MEFs were infected with 0.1 MOI of VSVΔM51-GFP virus for 24 h, and then cells were imaged by fluorescence microscopy (A) and the supernatants were collected and virus titer was measured by plaque assay (B).The data in B is from one representative experiment of three independent experiments (means ± SD of duplicate assays). A two-tailed Student’s t test was used to analyze statistical significance, * P < 0.05 versus the control groups. (PDF) [file ppat.1004783.s006.pdf]

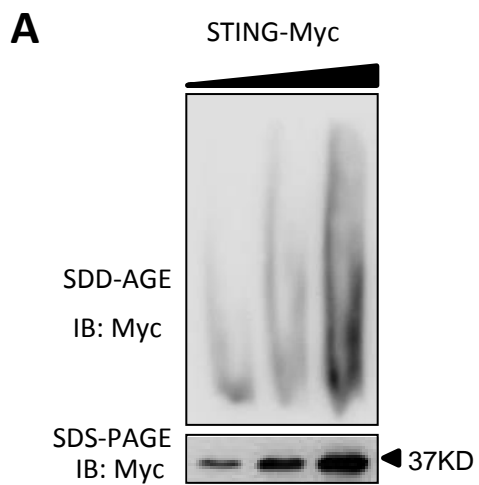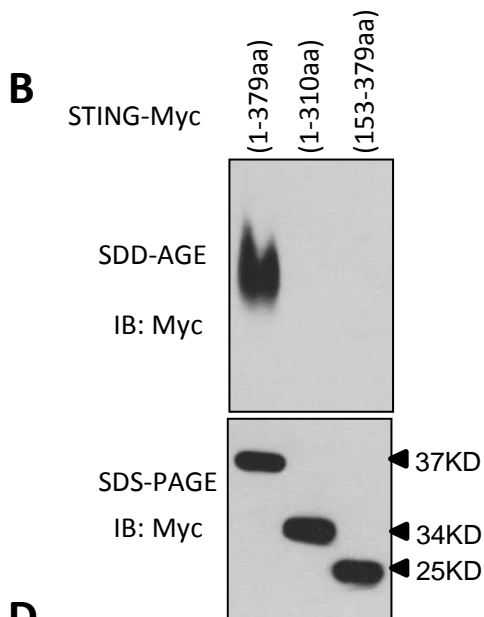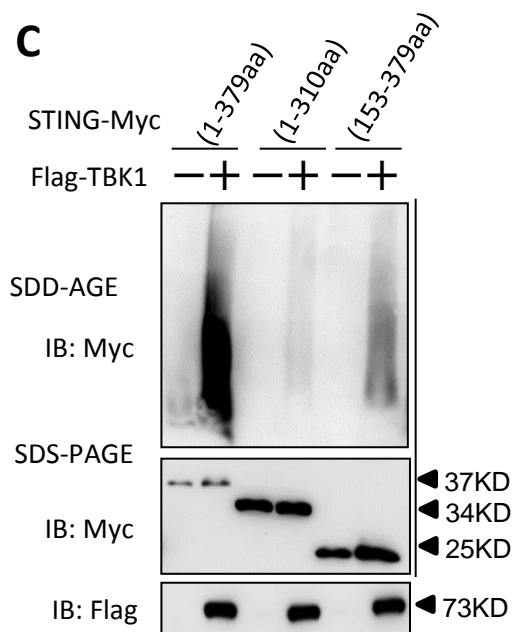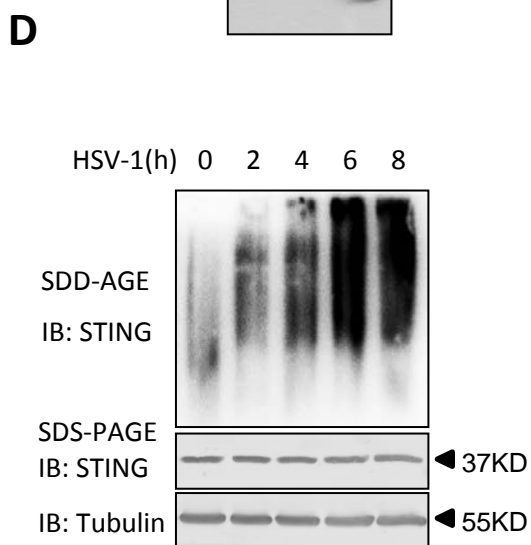

Supplement: S6 Fig — (A) STING formed high-molecular-weight aggregates in a dose-dependent manner. HEK293 cells were transfected with increased dose of STING-Myc (0.1 μg, 0.5 μg, 1 μg), and 24 h later, the cell lysates were resolved with SDD-AGE (upper panel) or SDS-PAGE (lower panel) and analyzed by immunoblotting with the indicated antibodies. (B) Only the full length of STING could form aggregates. HEK293 cells were transfected with STING-Myc (amino acids 1–379), STING-Myc (amino acids 1–310) or STING-Myc (amino acids 153–379), 24 h later, cells were lysed and analyzed by immunoblotting with the indicated antibodies. (C) Both the N- and C-terminal of STING are important for its aggregates induced by TBK1. HEK293 cells were transfected with STING-Myc (amino acids 1–379), STING-Myc (amino acids 1–310) or STING- Myc (amino acids 153–379) together with empty vector or Flag-TBK1, 24 h later, the cell lysates were resolved with SDD-AGE (upper panel) or SDS-PAGE (lower panel) and analyzed by immunoblotting with the indicated antibodies. (D) THP-1 cells were infected with 1 MOI of HSV-1 as indicated times and cells were lysed for SDD-AGE or SDS-PAGE assay. (PDF) [file ppat.1004783.s007.pdf]

**A****ISRE**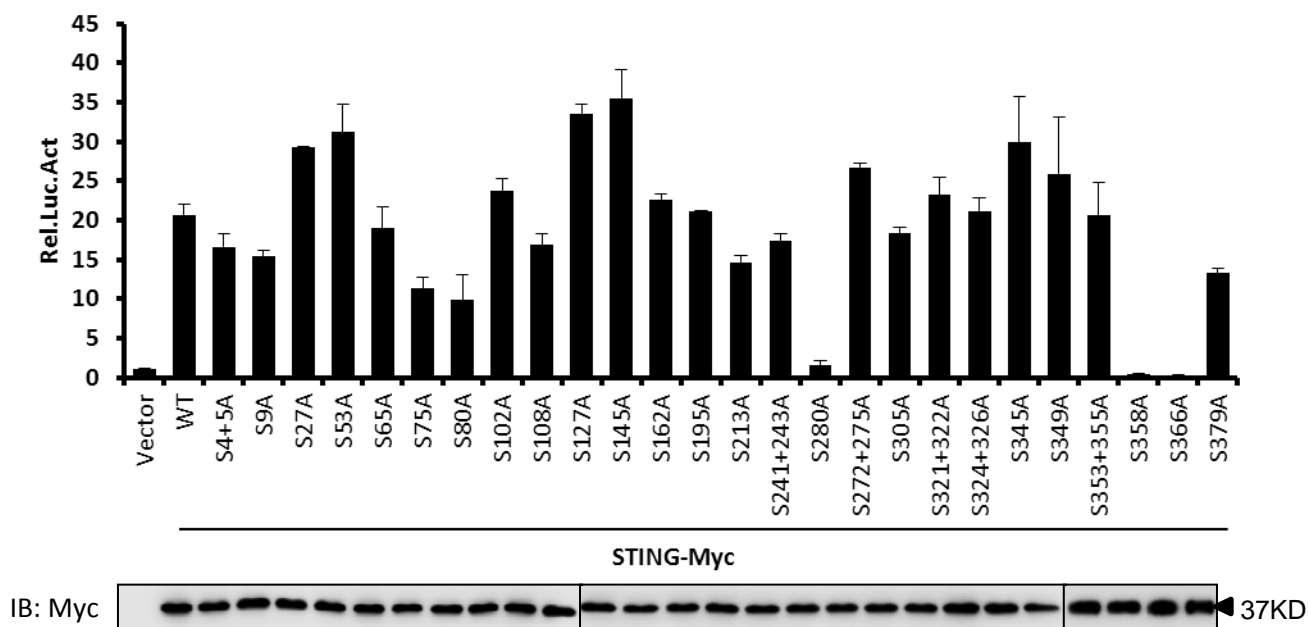**B****ISRE**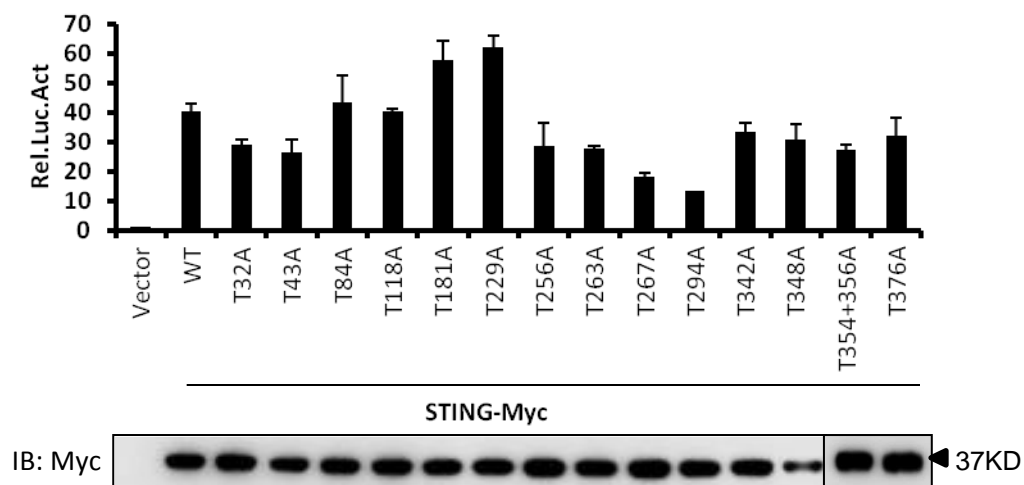

C

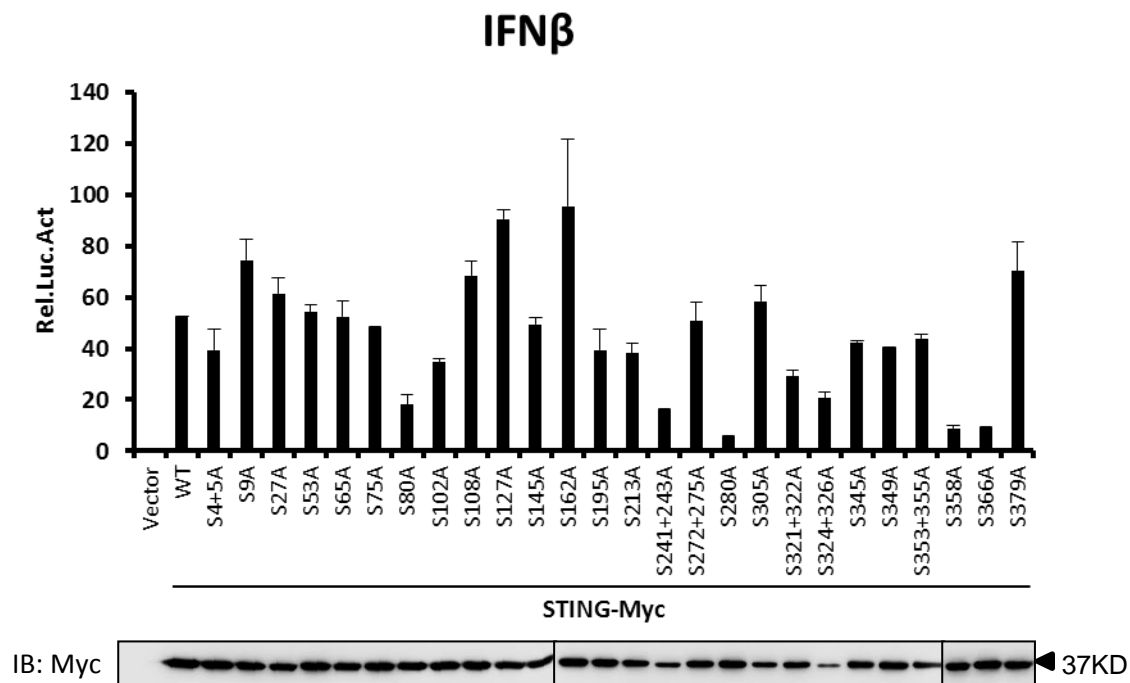

D

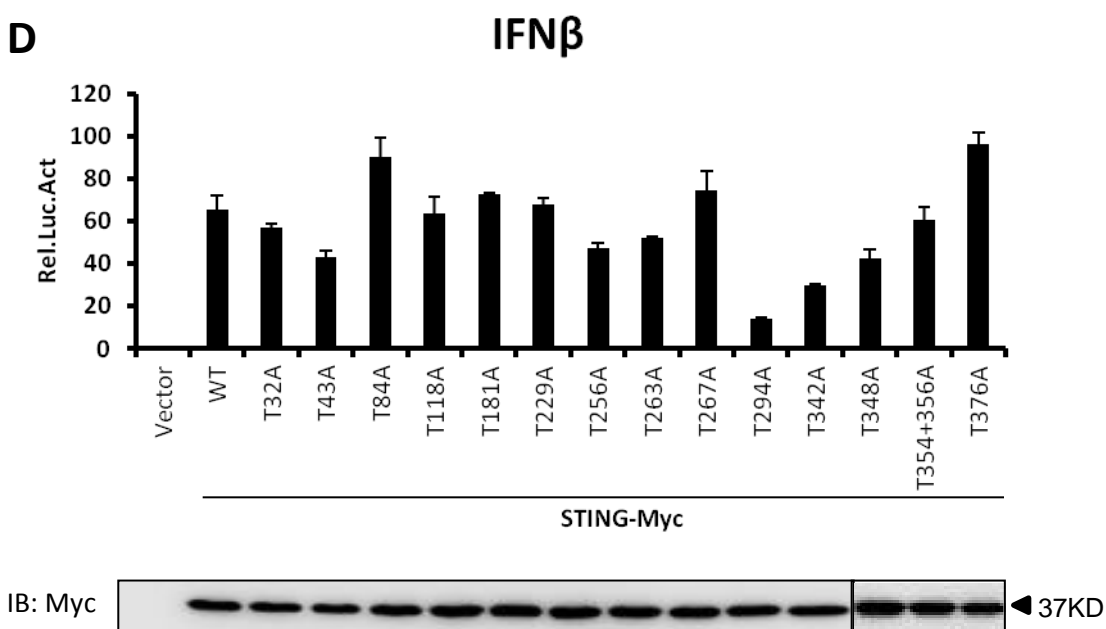

Supplement: S7 Fig — (A-D) Identification of STING Ser/Thr residues which are required for the activation of ISRE (A, B) and IFNβ (C, D) promoters. HEK293 cells were transfected with vectors encoding the indicated STING WT and mutants. At 30 h after transfection, the cells were lysed for luciferase assays (upper panel) and immunoblotting assays (lower panel). (PDF) [file ppat.1004783.s008.pdf]

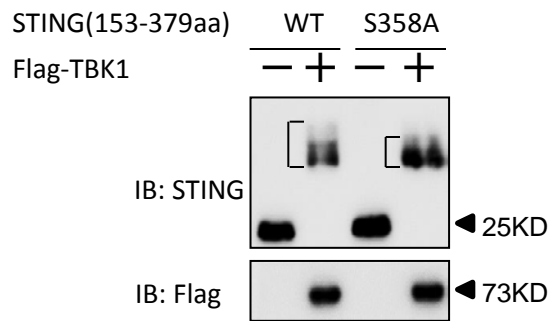

Supplement: S9 Fig — Purified His-STING-WT (amino acids 153–379,1 μg) or its mutant STING-S358A(1 μg) and Flag-TBK1 (1 μg) were incubated in kinase buffer for 30 min at 30°C and then analyzed by immunoblotting with the indicated antibodies. (PDF) [file ppat.1004783.s010.pdf]

**A**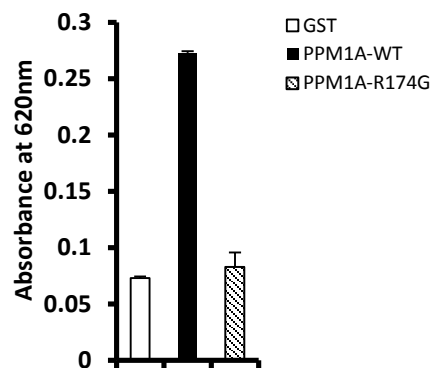**B**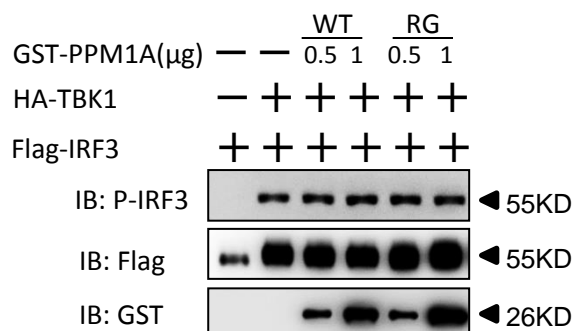**C**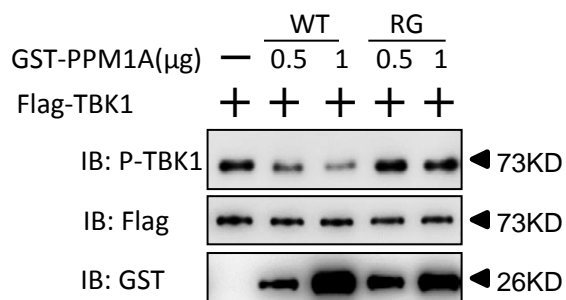**D**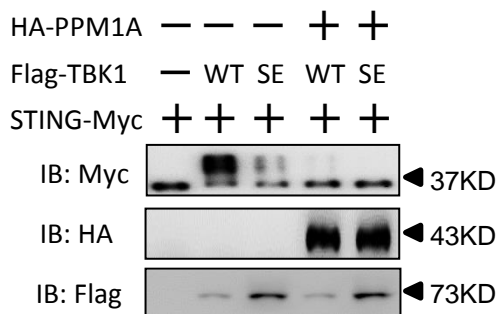

Supplement: S10 Fig — (A) Phosphopeptide assay was analyzed by Malachite phosphatase green assay. Synthesized STING phosphopeptide corresponding to phospho-S358 peptide was used as substrate, and incubated with equal amounts of GST, GST-tagged PPM1A-WT or PPM1A-R174G. The reaction was carried out for 60 minutes at 30°C and free phosphate was measured by malachite green assay. (B-C)TBK1, but not IRF3, was a direct target of PPM1A in an in vitro phosphatase assay. HEK293 cells were first transfected TBK1 alone (C) or in combination with IRF3 (B), respectively, and then lysed for co-IP experiments to purify the phosphorylated TBK1 and IRF3 proteins by the Flag peptides. Purified phosphorylated Flag-IRF3 (1 μg) (B) or Flag-TBK1(1 μg) (C) with the increased dose (0.5 μg, 1 μg) of GST-PPM1A-WT or GST-PPM1A-R174G were incubated in phosphatase buffer for 30 min at 30°C and then analyzed by immunoblotting with the indicated antibodies. (D) PPM1A dephosphorylated TBK1-WT- or TBK1-S172E-induced STING phosphorylation. HEK293 cells were transfected with plasmids as indicated, 24 h later, the cell lysates were resolved with SDS-PAGE and analyzed by immunoblotting with the indicated antibodies. The data in A is from one representative experiment of three independent experiments (means ± SD of triplicate assays). (PDF) [file ppat.1004783.s011.pdf]

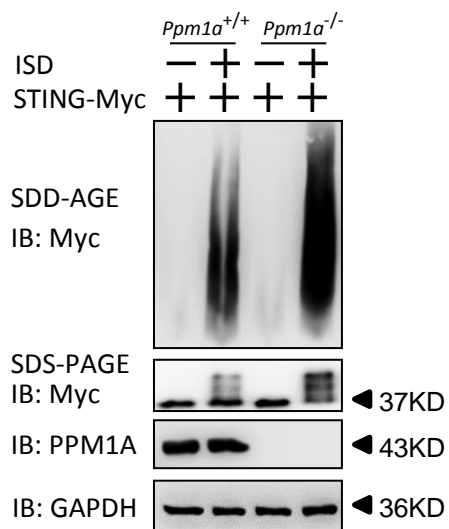

Supplement: S11 Fig — Ppm1a +/+ and Ppm1a -/- MEFs were first infected with lentivirus expressing STING-Myc, 36 h post-infection, cells were transfected with or without ISD(2 μg/ml) for 6 h, and then lysed for SDD-AGE or SDS-PAGE assay. (PDF) [file ppat.1004783.s012.pdf]

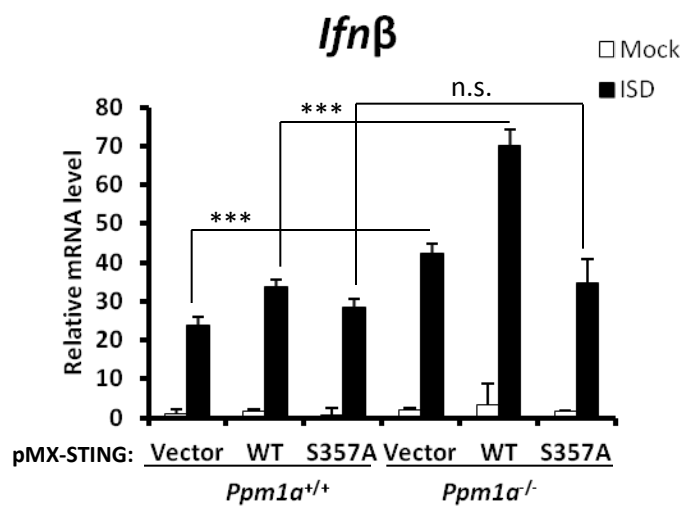

Supplement: S12 Fig — Ppm1a +/+ and Ppm1a -/- MEFs were first infected with retrovirus expressing STING-WT, STING-S357A or empty vector, at 48 h post-infection, cells were then transfected with ISD(3 μg/ml) for 7 h and followed by qRT-PCR analysis. Data shown are the relative abundance of Ifnβ to control groups. The data is from one representative experiment of three independent experiments (means ± SD of triplicate assays). A two-tailed Student’s t test was used to analyze statistical significance. n.s., No Significance; *** P < 0.001 versus the control groups. (PDF) [file ppat.1004783.s013.pdf]

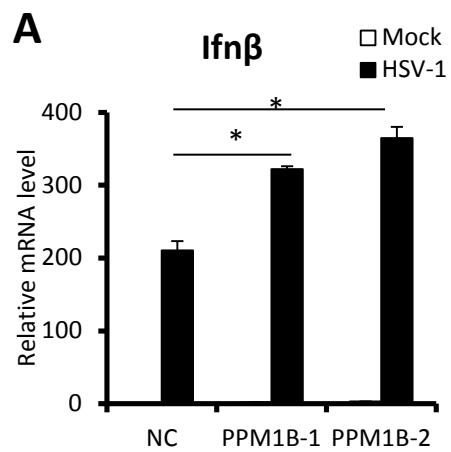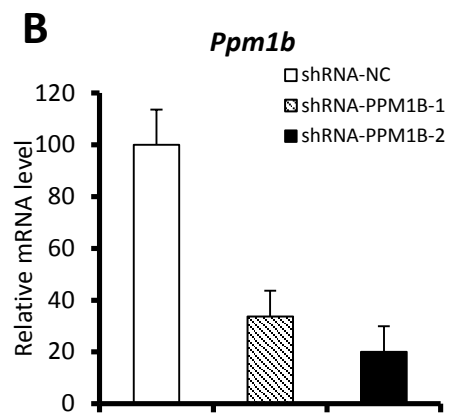

Supplement: S13 Fig — (A-B) THP-1 cells were first infected with a lentivirus targeting PPM1B for knockdown or NC for 72 h and then cells were infected with 1 MOI of HSV-1for 6 h and total RNA were isolated for qRT-PCR analysis. Relative mRNA levels of IFNβ (A) and PPM1B (B) were normalized to the GAPDH RNA levels in each sample. The IFNβ mRNA abundance of the control group (shRNA-NC, mock) was assigned a value of 1. The PPM1B mRNA abundance of the control group (shRNA-NC) was assigned a value of 100. The data in A and B is from one representative experiment of three independent experiments (means ± SD of triplicate assays in A and B). A two-tailed Student’s t test was used to analyze statistical significance, * P < 0.05, versus control groups. (PDF) [file ppat.1004783.s014.pdf]

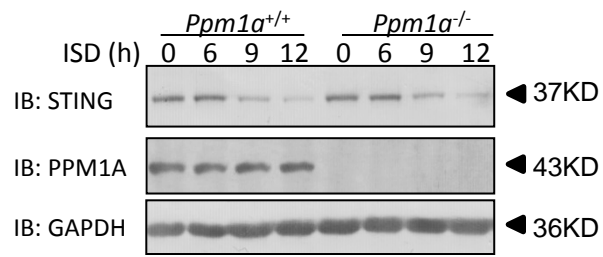

Supplement: S14 Fig — Ppm1a +/+ and Ppm1a -/-MEF cells were transfected with ISD (4 μg/ml) as indicated times and cells were collected and lysed for SDS-PAGE assay. (PDF) [file ppat.1004783.s015.pdf]
