# Supplementary material for: PPM1A Regulates Antiviral Signaling by Antagonizing TBK1-Mediated STING Phosphorylation and Aggregation
Source: PLoS Pathog. 2015 Mar 27;11(3):e1004783. doi: 10.1371/journal.ppat.1004783 (PMC4376777; doi:10.1371/journal.ppat.1004783)
Supplement: S8 Fig — (A)The localizations of identified phosphorylation sites by mass spectrometry are shown, together with the structure of STING, in which cytoplasmic, membrane and non-cytoplasmic regions of STING were annotated by InterProScan (http://www.ebi.ac.uk/interpro/search/sequence-search). The blue rectangle represents non-cytoplasmic domain, the red rectangle represents transmembrane region, and the green rectangle represents cytoplasmic domain. (B)The representative identified fragment ions, including b and y ions, from MS/MS spectra for S358 site of STING. “ph” means phosphorylation. Specific y ions surrounding phosphorylated S358 were identified, indicating a reliable identification. (C)Sequence alignment for STING protein sequences showed evolutionary conservation of S358 among mammals. (PDF) [file ppat.1004783.s009.pdf]

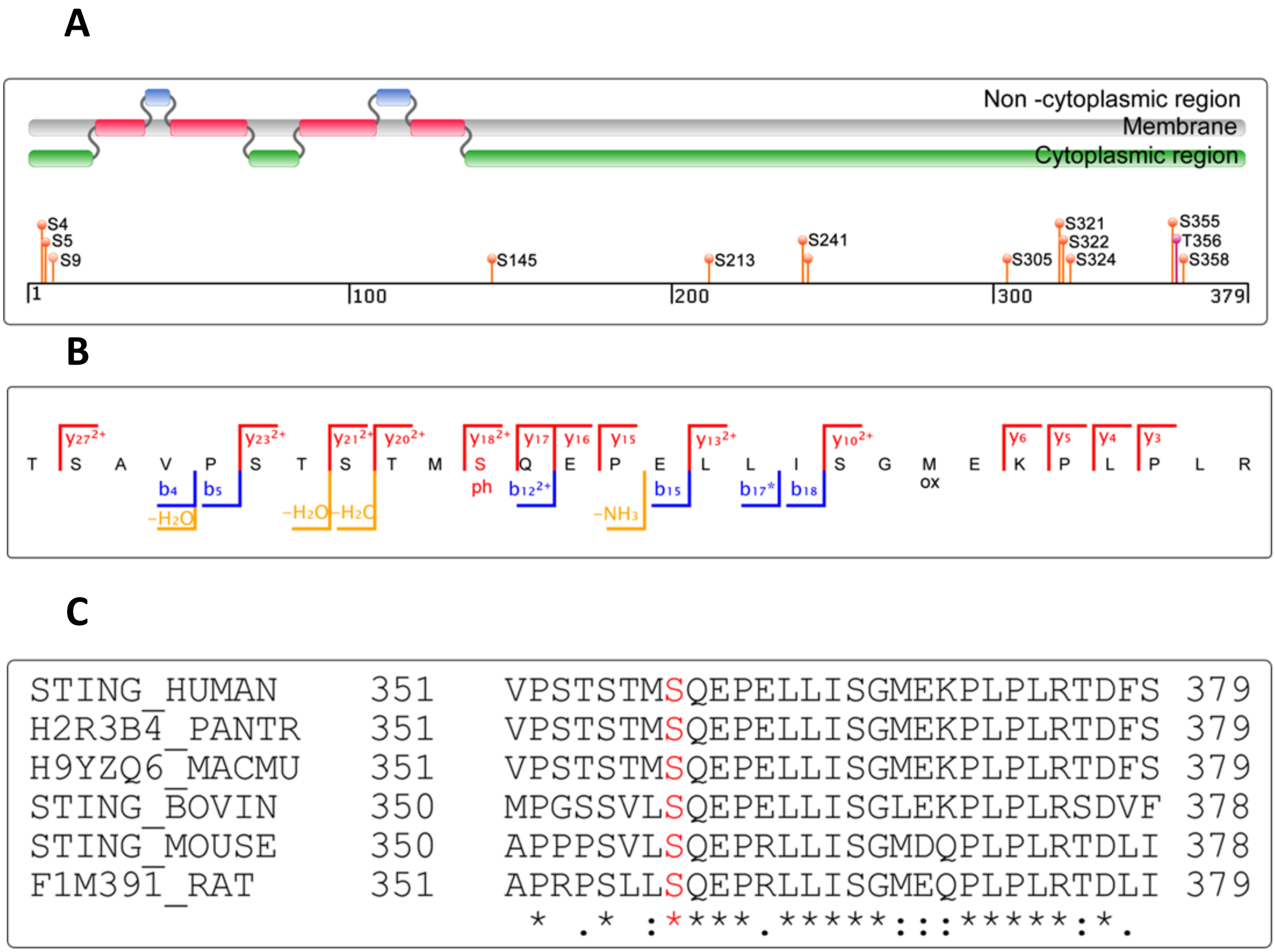
A
Non -cytoplasmic region
Membrane
Cytoplasmic region
S4
S5
S9
S145
S213
S241
S305
S321
S322
S324
S355
T356
S358
1
100
200
300
379
B
T S A V P S T S T M S Q E P E L L I S G M E K P L P L R
ph
ox
C
STING_HUMAN 351 VPSTSTMSQEPELLISGMEKPLPLRTDFS 379
H2R3B4_PANTR 351 VPSTSTMSQEPELLISGMEKPLPLRTDFS 379
H9YZQ6_MACMU 351 VPSTSTMSQEPELLISGMEKPLPLRTDFS 379
STING_BOVIN 350 MPGSSVLSQEPELLISGLEKPLPLRSDVF 378
STING_MOUSE 350 APPPSVLSQEPRLLISGMDQPLPLRTDLI 378
F1M391_RAT 351 APRPSLLSQEPRLLISGMEQPLPLRTDLI 379
